# Mechanochemical coupling of MGF mediates periodontal regeneration

**DOI:** 10.1002/btm2.10603

**Published:** 2023-10-07

**Authors:** Ying Zhao, Songbai Zhang, Bo Cheng, Fan Feng, Yue Zhu, Yanli Liu, Junjun Wang, Dehui Zou, Heng Ma, Feng Xu, Min Zhang

**Affiliations:** ^1^ State Key Laboratory of Oral & Maxillofacial Reconstruction and Regeneration, National Clinical Research Center for Oral Diseases, Shaanxi International Joint Research Center for Oral Diseases, Department of General Dentistry and Emergency School of Stomatology, Fourth Military Medical University Xi’an People's Republic of China; ^2^ Department of Anesthesiology and Perioperative Medicine Xi'an People's Hospital (Xi'an Fourth Hospital), Northwest University Xi'an People's Republic of China; ^3^ The Key Laboratory of Biomedical Information Engineering of Ministry of Education School of Life Science and Technology, Xi'an Jiaotong University Xi’an People's Republic of China; ^4^ Bioinspired Engineering and Biomechanics Center (BEBC), Xi'an Jiaotong University Xi’an People's Republic of China; ^5^ Department of Physiology & Department of Pathophysiology School of Basic Medical Sciences, Fourth Military Medical University Xi’an People's Republic of China

**Keywords:** Fyn/FAK signaling, mechanical stimulation, mechano‐growth factor (MGF), periodontal ligament stem cells (PDLSCs), periodontal regeneration

## Abstract

Clinical evidence shows that the mechanical stimulation obtained from occlusion could enhance periodontal ligament (PDL) remodeling. Mechano‐growth factor (MGF) is a growth factor produced specifically following mechanical stimulus Here, we aim to investigate the mechanical enhancement potential and mechanism of the MGF in PDL regeneration. In vivo study found that MGF produced from the PDL under occlusion force could strongly enhance PDL remodeling. In vitro experiments and mathematical modeling further confirmed the mechanical enhancement effect of MGF for PDLSC differentiation toward fibroblasts. A mechanochemical coupling effect of MGF mediated the enhancement of mechanical effect, which was modulated by Fyn‐FAK kinases signaling and subsequent MAPK pathway. Finally, enhanced PDL regeneration under the mechanochemical coupling of MGF and occlusal force was verified in vivo. There exists an additive mechanical effect of MGF mediated by Fyn‐FAK crosstalk and subsequent ERK1/2 and p38 phosphorylation, which could be developed as an MGF‐centered adjuvant treatment to optimize PDL remodeling, especially for patients with weakened bite force or destroyed periodontium.

**Abbreviations**
AOD: average optical density
EGFR: epidermal growth factor receptor
FAK: focal adhesion kinase
HGFR: hepatocyte growth factor receptor
IGF‐1: insulin‐like growth factors
IGF‐1R: insulin‐like growth factor receptor
MAPK: mitogen‐activated protein kinase
MGF: mechano‐growth factor
PDL: periodontal ligament
PDLSCs: periodontal ligament stem cells
PDGFR‐α/β: platelet‐derived growth factor receptor‐α/β
JNK1/2: c‐Jun N‐terminal kinase 1/2
ERK1/2: extracellular regulated protein kinases 1/2
FGFR1/2: fibroblast growth factor receptor 1/2
VEGFR2/3: vascular endothelial growth factor receptor 2/3

**Translational lmpact Statement**

As a growth factor under specific mechanical stimuli, MGF can promote the repair of force‐related tissues. In this study, we found that bite force stimulation can promote the expression of MGF in the periodontal membrane. The results provide the first evidence that MGF may enhance the mechanobiological effects on periodontal regeneration by mechanical effect. MGF is expected to be developed as an adjuvant treatment to maintain periodontal health in individuals with a weakened bite force or ruined periodontium by dental trauma or periodontitis.

## 1 INTRODUCTION

The periodontium, namely the periodontal ligament (PDL), is a specialized soft tissue in the body sandwiched between the hard tissues of teeth and bone that is characterized by its load‐bearing capacity.^1^ Under occlusal force, the metabolic activities of cells in PDL are triggered and activated, thus affecting PDL regeneration and remodeling.^2^ In the case of tooth avulsion, the entire PDL will be completely torn.^3^ The regeneration of PDL is a prerequisite for the normal function of teeth. Although tooth reimplantation is an effective therapy for tooth avulsion, the therapeutic effect after most PDL ruptures is still far from satisfactory clinically.^4^, ^5^ Clinical evidence has shown that the mechanical stimulation obtained from occlusion could enhance regeneration of the periodontium.^6^, ^7^, ^8^, ^9^ However, the underlying mechanism is not clear.

MGF is a growth factor that was first identified in mechanically stimulated skeletal muscle.^10^, ^11^ Subsequently, MGF was found in many other tissues, such as ischemic tissue of brain,^12^ stromal cells of the eutopic endometrium,^13^ malignancies,^14^ osteoblasts,^15^ growth plate chondrocytes^16^ et al. Studies have confirmed that MGF is involved in the repair and regeneration process of muscles,^17^ bones,^18^ nerves,^19^ tendons,^20^ cartilage,^16^ and many other tissues and organs under biomechanical environments after damage. It even showed outstanding performance in enhancing skeletal muscle movement.^21^ Here, the study was the first to focus on the MGF expression in periodontal ligament. Periodontium is highly sensitive to mechanical stimulation and do endure mechanical stimulation for a long time. Our study is expected to be developed as an adjuvant treatment to optimize periodontium regeneration in individuals with ruined periodontium by dental trauma or a weakened bite force (such as long‐term astronauts in orbit, elderly people with Alzheimer's disease, patients with dysfunction of the temporomandibular joint or mandibular muscle due to tumor or trauma). As the enhancement phenomena are quite common in living organisms (e.g., transcription, metabolism, and molecular assembly), we speculated that the upregulated MGF may be linked to the enhanced regeneration of the periodontium by mechanical stimuli. Thus, additive mechanical effects may also exist. However, the involved mechanisms remain elusive.

Mechanical stimuli activate multiple kinases within the focal adhesion (FA) complex at the plasma membrane including focal adhesion kinase (FAK) and Src family kinases (SFKs).^22^ Fyn, a member of the Src family of kinases, regulates cell proliferation and migration during tissue regeneration and transformation, implicating its conserved and specific functions.^23^ FAK is closely related to mechanical stimuli, which plays a key role in regulating collagen fibrillogenesis and fibroblast differentiation in PDL.^24^ FAK functions cooperatively with SFKs during mechanical signaling, and mechanical stimuli may increase the Src‐FAK association.^25^ The signaling of these tyrosine kinases might not be transduced by the same pathway under mechanical stimuli, but its underlying mechanisms require further investigation. Our study confirmed that MGF and hydraulic pressure may upregulate Fyn and FAK phosphorylation respectively in PDLSCs. As such, we wonder whether MGF could strengthen the mechanobiological effects through specific mechanotransduction signaling, so as to realize the enhancement through mechano‐chemical coupling. We hypothesized that Fyn and FAK may function in the additive mechanical effect of MGF.

Here, we established an animal model by feeding powder/block food to observe the mechanical enhancing effects of occlusal loading on the regeneration of PDL and MGF expression in it. The effects of MGF and mechanical stimuli on PDLSCs differentiation and the possible signaling pathways that potentially mediate the observed effects in vivo were explored in vitro. We studied the influences of the additive mechanical effect of MGF in delayed replantation of avulsed teeth for bite force adjustment rats, and further verified that MGF could strengthen the mechanobiological effects in delayed replantation of avulsed teeth for bite force adjustment rats. Overall, our results provide the first evidence that MGF may enhance the mechanobiological effects on periodontium regeneration by additive mechanical effect. The results are expected to be developed as an adjuvant treatment to optimize periodontium regeneration in individuals with a weakened bite force or ruined periodontium by dental trauma.

## 2 MATERIALS AND METHODS

### 2.1 PDL depth evaluated by using micro‐CT

**FIGURE 1:**
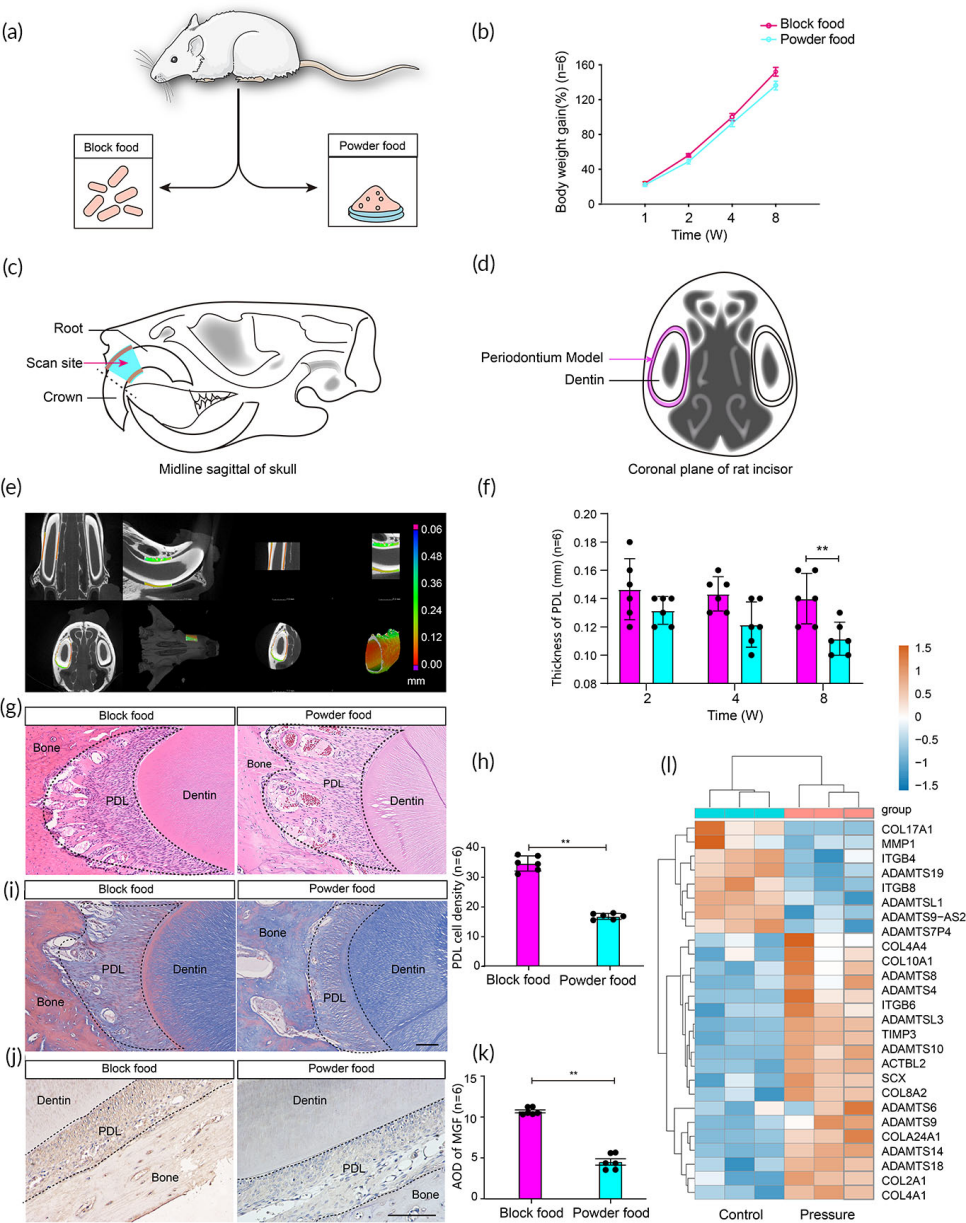
Occlusal force‐derived mechanical stimulation is critical for the maintenance of PDL architecture and MGF expression. (a) Occlusal force was adjusted by feeding the animals block food and powder food. (b) The weight of the rats in the block food group and the powder food group continued to increase as the feeding time increased, but no statistically significant difference was not observed between the two groups. (c) Schematic diagram from sagittal plane of Micro‐CT scanning of the rat maxilla. The scan site of tooth root area was selected at 1 mm close to the internal tooth margin for reconstruction. (d) Schematic diagram from coronal plane of Micro‐CT scanning of the rat maxilla. One hundred and fifty scanned pictures were selected to establish the periodontium model. (e) Micro‐CT Scanning was performed perpendicular to the long axis of the tooth. Horizontal sections and coronal sections of Micro‐CT scan highlighted upper central incisors and PDL structures. 3D reconstruction of PDL of the maxillary central incisors of rats were obtained using micro‐CT. The color bars represent the thickness of PDL (mm). (f) Comparison of the average thickness of the periodontium in the powder food and block food groups captured by micro‐CT scanning. (g) HE staining of the maxillary central incisors in the powder food and block food groups. (h) Statistical analysis of the PDL cell density in the powder food and block food groups. (i) Masson's trichrome staining of the maxillary central incisors in the powder food and block food groups. (j) Immunohistochemical examination of MGF expression in PDL. (k) Comparison of the intensity of immunohistochemical staining for MGF. All assessments were made on the PDL region of the specimen. (l) Heatmap of published gene expression omnibus (GEO) data showing Scleraxis, Collagens, Integrins and matrix metalloproteinases expressions in intermittent compressive force treated PDL and untreated PDL.^31^ (**p < 0.01, vs. the block food group, *n* = 6).

Twenty‐four male SD rats (200–250 g) obtained from the Animal Center (Fourth Military Medical University) were divided into two groups to observe the periodontium depth of rat incisors. Animal studies were confirmed with the ARRIVE guidelines 2.0 (https://arriveguidelines.org/arrive-guidelines). The mathematical formula $n=\left(\frac{{\left(\mathrm{Z\alpha}+\mathrm{Z\beta}\right)}^{2}\times 2\sigma^{2}}{\delta^{2}}\right)$ was used to confirm the sample number. Here, n indicates the sample number; α as 0.05; β as 0.9. From the data we got from the similar reports, we confirmed the σ and δ,^26^, ^27^ σ as standard deviation; δ as the difference between mean values for two groups. Thus, the sample number of rats needed for validating each parameter in the present study was 6 for immunofluorescence and micro‐CT scanning. It is reported that sex hormones can affect wound‐healing process. Also, the periodontal health status is profoundly affected by hormone levels. Therefore, to exclude the effects of estrogen in different sex, only the male rat we adopted in the present study for the periodontal healing observation. The BF group was provided block food (AIN‐76A, Moldiets, China), and the PF group was provided powdered food (AIN‐76A, Moldiets, China; Figure 1a). Rats were sacrificed at 2, 4, and 8 weeks, and the maxilla was collected for detection. The long axis of the tooth was maintained perpendicular to the scanning plane during micro‐CT scanning (Y. XLON, Germany). The scanning area was located from the edge of the central incisor to the apex of the tooth. The following parameters were set: the interlaminar spacing was 20 μm, the voltage was 90 kV, the current was 50 μA, the projection number was 720, and the exposure time was 0.6 s. After image scanning was completed, VG Studio MAX 3.0 software was used to reconstruct the three‐dimensional image. The tooth root area was selected at 1 mm close to the internal tooth margin for reconstruction, 150 scanned pictures were selected to establish the periodontium model, and the average thickness of the periodontium was recorded. The scanned reconstruction area of the PDL was marked with color for observation and measurement.

### 2.2 MGF expression in PDL detected by immunohistochemistry assay

Animals were grouped and treated as described above. After micro‐CT scanning, the maxilla blocks were decalcified in 10% EDTA at 4°C for 4 weeks, followed by dehydration, paraffin embedding, and sectioning. Tissue sections with a thickness of 6 μm were prepared. The sections were stained with hematoxylin–eosin and Masson's trichrome stain for microscopic observation. Images of the histological sections were obtained using a Leica 5000B microscope (Leica Microsystems, Germany). We noted the change in cell nuclei and performed a digital image analysis using Image‐Pro Plus 6.0 software (Adobe Systems Software Ireland Ltd., CA, USA) to confirm the change in cell density in the periodontal ligament. Other histological changes are detailed in the Supplementary Material (Figure S1).

Next, immunohistochemical staining was performed as described below. Rat incisor tissues were washed with cold phosphate‐buffered saline (PBS, pH 7.4) immediately and fixed with 4% paraformaldehyde. After 24 h of postfixation at 4°C, tissues were decalcified in 10% EDTA at 4°C for 4 weeks and then dehydrated and embedded in paraffin. Tissues were sectioned sagittally at a thickness of 6 μm. Next, sections were incubated in boiling 0.01 mol/L citrate buffer (pH 7.2) for 10 minutes, cooled to room temperature, immersed in 3% H_2_O_2_ in methanol for 10 min, and washed with PBS (0.01 mol/L, pH 7.2) 3 times. Subsequently, 10% normal goat serum was added and incubated for 1 h. The sections were incubated with a primary anti‐MGF antibody (Phoenix Biotech, Catalog H‐033‐35, USA) overnight at 4°C. Sections were washed with PBS (0.01 mol/L, pH 7. 2) and incubated with anti‐rabbit IgG‐horseradish peroxidase (HRP) for 2 h at room temperature (approximately 25°C). Images of the histological sections were obtained using a Leica 5000B microscope (Leica Microsystems, Germany). Images of immunohistochemical staining were analyzed using Image‐Pro Plus 6.0 software to determine the average optical density (AOD) values of MGF expression. All assessments of MGF were made on the PDL region of the specimen.

### 2.3 Effects of MGF and cyclic hydraulic pressure on the cytobiological characteristics of PDLSCs

PDLSCs were isolated and purified according to previously study.^28^ Briefly, human impacted third molars were extracted from 3 systemically healthy adults (18–30 years of age) at the School of Stomatology, Fourth Military Medical University (FMMU). The teeth were immediately immersed into an ice‐cold phosphate‐buffered saline (PBS; Hyclone, Road Logan, UT, USA) solution that contained 100 U/mL penicillin/streptomycin (Sigma‐Aldrich, St. Louis, MO, USA) and transferred to the laboratory. The protocols were approved by the Institutional Research Review Board at the School of Stomatology of FMMU (No. IRB‐REV‐2022128) and that the experiments were performed in accordance with the Declaration of Helsinki (2008) for humans. Isolation, purification, and identification of PDLSCs were described in detail in the Supporting Information and Figure S2. PDLSCs were cultured in dishes subjected to the periodic dynamic pressure, which was exerted by the multifunctional hydraulic cellular pressure unit reported in our previous studies.^29^, ^30^

**FIGURE 2:**
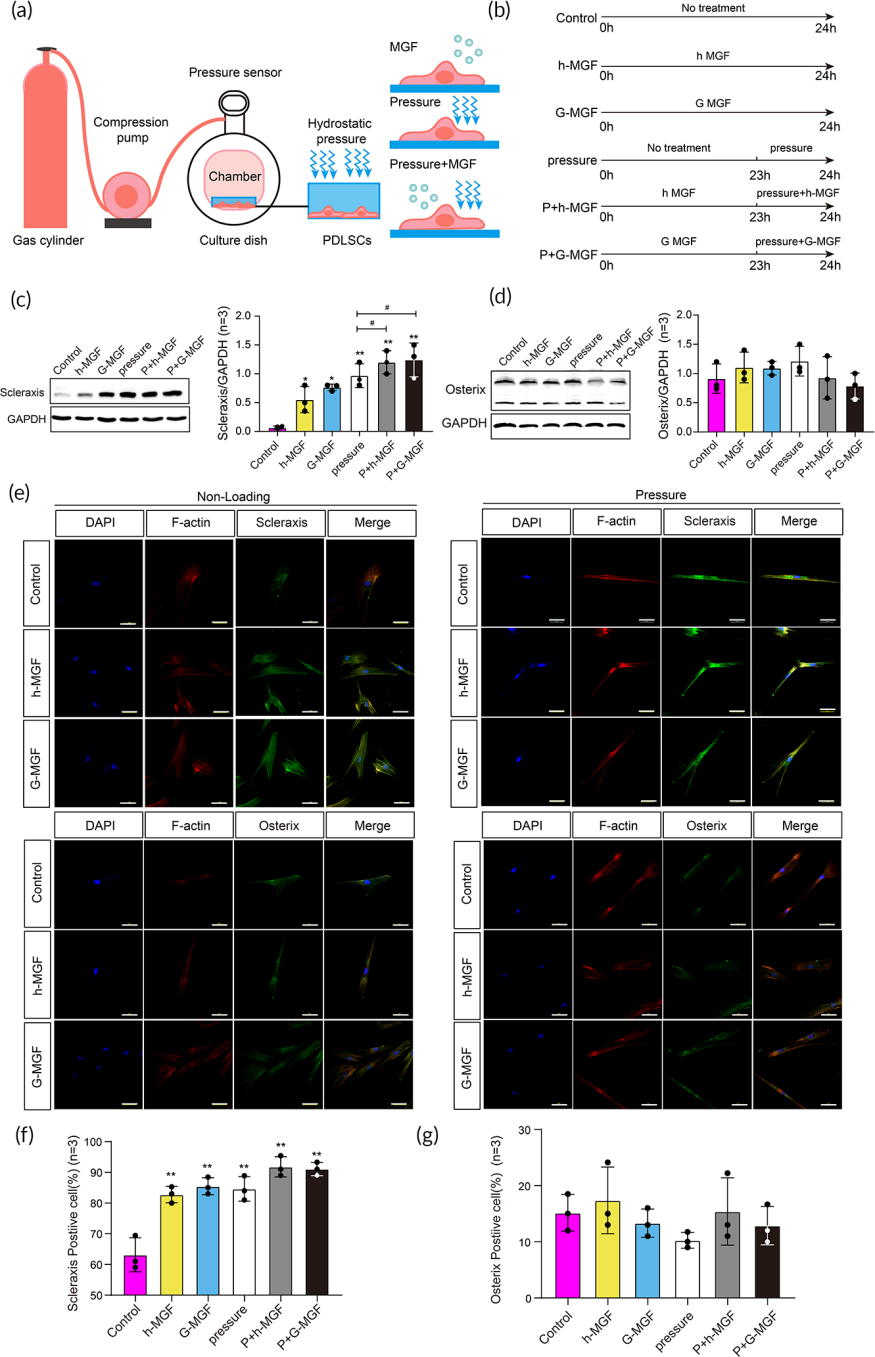
MGF potentiates the mechanobiological effect of PDLSC differentiating toward fibroblasts. (a) Illustration of PDLSC treatment in vitro. (b) Schematic diagram of grouping and the different treatments applied. (c) The protein level of the Scleraxis quantified using Westerns blotting. (d) The level of the Osterix protein was quantified using Western blotting. (e) Confocal immunofluorescence images of Scleraxis and Osterix expression in different groups (bar = 50 μm). (f, g) Quantitative results of the ratio (%) of Scleraxis and Osterix positive cells (**p < 0.01, vs. control group, ^#^ *p* < 0.05, compared with the pressure group, *n* = 3).

The PDLSCs were assigned to 6 groups: the control group (ɑ‐MEM without FBS), h‐MGF group (ɑ‐MEM with 30 ng/mL h‐MGF), G‐MGF group (ɑ‐MEM with 30 ng/mL of G‐MGF), pressure group (hydraulic pressure of 0–120 kPa for 1 h, 0.1 Hz), P + h‐MGF group (hydraulic pressure and 30 ng/mL of h‐MGF) and P + G‐MGF group (hydraulic pressure and 30 ng/mL of G‐MGF). The specific grouping was as follows: In control group, PDLSCs were maintained in serum‐free condition. In h‐MGF or G‐MGF treatment group, cells were treated with MGF for 24 h. In Pressure + h‐MGF or Pressure + G‐MGF group, MGF was also added for 24 h, with the cells being treated with hydraulic pressure (0–120 kPa) for the last hour. The time schedule for the different treatment groups was shown in Figure 2b. To exclude the potential effects of cytokines in serum, PDLSCs in all groups were maintained in serum‐free condition.

Since the differentiation direction of PDLSCs is very important for determining the ideal periodontal healing or pathological ankylosis (root‐bone adhesion) of PDL after avulsion, we detected the fibroblast and osteogenic differentiation markers using Western blotting and immunofluorescence double‐staining. The total proteins were extracted from the different groups with a Total Protein Extraction Kit with protease inhibitors and phosphatase inhibitors (Beyotime Biotechnology, China). Protein concentrations were measured using the BCA Protein Quantitation Kit (Beyotime Biotechnology, China). Equal amounts of protein were separated on 10% sodium dodecyl sulfate‐polyacrylamide gel electrophoresis (SDS–PAGE) gels (Cwbiotech, China) and electrotransferred onto polyvinylidene fluoride (PVDF) membranes (Millipore, USA). The membranes were blocked with Tris‐buffered saline (100 mM NaCl; 10 mM Tris–HCl, pH 7.5; 0.1% Tween 20) containing Tween‐20 and 5% nonfat dry milk at room temperature for 1 h. The blots were incubated with a rabbit polyclonal anti‐Scleraxis antibody (1:1000; (Abcam Cat# ab58655, RRID: AB_882467) and rabbit monoclonal anti‐Osterix antibody (1:1000; Santa Cruz Biotechnology Cat# sc‐393325, RRID: AB_2895257) overnight at 4°C, followed by incubation with a rabbit anti‐goat antibody (1:10,000; Abcam, England) at room temperature for 1 h. Immunodetection was performed using an ECL chemiluminescence detection kit (Thermo, USA), and the integrated optical density (IOD) values of each blot were analyzed using Gel Pro software, version 4.0 (Media Cybernetics, USA). GAPDH (1:1000 Abcam, England) was analyzed as a reference protein.

Next, the spatial expression of Scleraxis was observed using immunofluorescence staining. PDLSCs were seeded on polylysine‐coated slides and incubated for 24 h (1 × 10^4^ cells/well, NUNC, USA). After treatment, the cells were fixed with 4% paraformaldehyde for 10 min and incubated with PBS containing 0.25% Triton X‐100 for 10 min. Afterward, the cells were blocked and incubated with a rabbit polyclonal anti‐Scleraxis antibody (1:1000; Abcam Cat# ab58655, RRID: AB_882467) and rabbit monoclonal anti‐Osterix antibody (1:1000; Santa Cruz Biotechnology Cat# sc‐393325, RRID: AB_2895257) overnight at 4°C. Samples were subsequently incubated with biotinylated horse anti‐mouse immunoglobulin G and goat anti‐rabbit immunoglobulin G (1:100 Zhongshan Biotechnology, Beijing, China) as secondary antibodies. The actin cytoskeleton was assessed by staining with phalloidin‐Alexa Fluor 594 (red) (5 μg/mL, Molecular Probes, USA), and nuclei were stained with DAPI (blue; Invitrogen, USA). Images were captured using a Nikon C2 Confocal microscope (Nikon, Japan).

### 2.4 Molecular signaling pathway initiated by MGF and mechanical pressure in PDLSCs

The PDLSCs were assigned as described in the previous section (Figure 2). The phosphor RTK‐antibody array was obtained from Raybiotech, Inc. Cells in different groups were lysed using lysis buffer (Raybiotech Inc., USA) supplemented with 1 mM phenylmethylsulfonyl fluoride. Lysates were analyzed using the phosphor RTK‐antibody microarray. The array consisted of 71 antibodies. Briefly, 250 μg/mL of cell lysates (1 mL) were added to the incubation wells with the antibodies overnight at 4°C. After washing the wells several times, HRP‐streptavidin was added to each well and incubated for 2 h at room temperature. Immunodetection was performed using the Image Quant LAS4000 System (General Electric Company, USA). All procedures were performed according to the manufacturer's instructions. Positive and negative controls for each protein were used to normalize the results. The densitometry data of each array spot were quantified and used to calculate the mean value.

Next, protein extraction and quantification were performed as described previously using the Total Protein Extraction Kit (Beyotime Biotechnology, China) and BCA Protein Quantitation Kit (Beyotime Biotechnology, China). The protocols for Western blotting were the same as described above. Rabbit polyclonal antibodies against FAK (Cell Signaling Technology Cat# 3285, RRID: AB_2269034), phospho‐FAK (Tyr397) (Cell Signaling Technology Cat# 8556, RRID: AB_10891442); Fyn Antibody (Cell Signaling Technology Cat# 4023, RRID: AB_10698604); phosphor‐Fyn Antibody (Abcam Cat# ab188319, RRID: AB_2938855); AKT antibody (Cell Signaling Technology Cat# 9272, RRID: AB_329827), phosphor‐Akt (Ser473) (Cell Signaling Technology Cat# 4060, RRID: AB_2315049), ERK1/2 (Cell Signaling Technology Cat# 4695, RRID: AB_390779), phosphor‐ERK1/2 (Thr202/Tyr204) (Cell Signaling Technology Cat# 4370, RRID: AB_2315112), SAPK/JNK Antibody (Cell Signaling Technology Cat# 9252, RRID: AB_2250373), Phospho‐SAPK/JNK (Thr183/Tyr185) (Cell Signaling Technology Cat# 4668, RRID: AB_823588), p38 (Cell Signaling Technology Cat# 8690, RRID: AB_10999090), and phosphor‐p38 (Thr180/Tyr182) (Cell Signaling Technology Cat# 4511, RRID: AB_2139682) were used as primary antibodies, followed by incubation with a rabbit anti‐goat secondary antibody (1:10,000; Abcam, England). Immunodetection and analyses were conducted as described above. GAPDH (1:1000, Abcam, Cat# ab8245) Anti‐rabbit IgG HRP‐linked Antibody (Cell Signaling Technology Cat# 7074, RRID: AB_2099233) was used as the reference protein.

For Src family kinase inactivation experiments, cells were treated with 4‐Amino‐5‐(4‐chlorophenyl)‐7‐(t‐butyl) pyrazolo[3,4‐d] pyrimidine (PP2), which was purchased from MedChemExpress (HY‐13805, Trenton, NJ, USA). According to the previous study, PP2 at a concentration of 10 μM inhibits Src activity in cultured cells, but having no inhibitory effect of cell proliferation.^31^ When the cells were treated with PP2, with a concentration of 10 μM was used at 37°C for 24 h. Afterward, PDLSCs of different groups were treated with pressure and/or MGF respectively according to the experimental design.

### 2.5 Gene expression of MGF in PDLSCs under pressure by real‐time PCR detection

PDLSCs were isolated and purified as described in a previous study.^28^ The method is detailed in the Supplementary Material (Figure S2). The multifunctional hydraulic cellular pressure unit previously reported in our series of studies was employed here.^30^ The device provided either hydrostatic or hydraulic pressure within a relatively large range and monitored every change in pressure and temperature inside the incubator in real time using monitoring software. Based on previous studies, periodic dynamic pressures of 0–90, 0–120, and 0–150 kPa were adopted here for the feasible screening of biomechanical conditions, with the frequency maintained at 0.1 Hz for 1 h.

Next, samples in each group were collected at 3 weeks of in vitro culture (*n* = 3 per group). Total RNA was extracted by using Trizol reagent (Takara, Japan) according to the manufacturer's instructions. The RNA pellets were reconstituted in DEPC‐treated water. cDNA was reversely transcribed from total RNA using a cDNA synthesis kit (Prime Script® RT reagent kit, Japan) according to the manufacturer's instructions. The mRNA expression levels of MGF were determined by real‐time PCR using SYBR Premix EX Taq (Takara, Japan). The sequences of primers used are the following: Mgf‐F: CGAAGTCTCAGAGAAGGAAAGG, Mgf‐R: ACAGGTAACTCGTGCAGAGC. Gapdh‐F: CGGAGTCAACGGATTTGGTCGTAT, Gapdh‐R: AGCCTTCTCCATGGTGGTGAAGAC. To normalize the mRNA levels, the GAPDH housekeeping gene was used as an internal control. Data were calculated as relative expression according to the 2^−ΔCT^ principle. These experiments were performed in triplicate and were repeated at least three times.

### 2.6 Mathematical modeling analysis for additive mechanical effect of MGF in PDLSCs

The model mainly captured the three‐potential regulation between molecules observed experimentally: (1) Pressure mediated the activation of FAKY397; (2) FAK‐p38 axis mediated the expression of Scleraxis, and (3) MGF‐Fyn‐ERK axis promotes the expression of Scleraxis. Since integrins are key molecules in such pressure mechanosensing, one key assumption is that we assumed that the relationship between activated FAKY397 level and mechanical stimuli (pressure here) is Hill‐type function. Similar relationship between substrate stiffness and FAKY397 level is used in our previous model. Here, we used the stochastic simulation^32^ to model the pressure and MGF‐mediated mechanical regulation in cells (Table S1). Since there is currently no experimental basis for the above relevant parameters. Thus, we adjusted all rate constants to fit our experimental observations, that is, Scleraxis presents a maximum value at 12–24 h and decreases to a normal level at 36–40 h. Similarly, for simplify, molecular number of FAK and MGF proteins are set 1000 and 100, the same order of magnitude as Rho GEF.^33^ Actually, the main conclusions drawn from the model were insensitive to the values chosen for rate constants or initial molecular number or rate constant. Models were solved numerically using Matlab (The Mathworks, Natick, MA, USA).

### 2.7 In vivo PDL regeneration under coupling of occlusal loading and MGF

Twenty‐four male SPF SD rats (200–250 g) obtained from the Animal Center (Fourth Military Medical University) were used to create a delayed replantation model of avulsed teeth as the animal model of periodontium regeneration with occlusal force regulation. Two different occlusal forces were simulated by feeding rats AIN‐76A block (Moldiets, China) and AIN‐76A powder. The right maxillary incisors were adopted as the experimental teeth position. After general anesthesia, the right maxillary incisors were carefully extracted from each rat. Unlike humans incisors, the rodent incisors can grow continuously throughout life, due to the existence of dental stem cell populations located in the apical end of the tooth.^34^ To avoid any interference and possible side‐effects on the results of the study, we removed the apical papilla of the rat teeth as previously described in a number of studies.^35^, ^36^ Animal studies confirmed with the ARRIVE guidelines 2.0. The mathematical formula *n* = $\varphi^{2}\left(\sum S_{i}^{2}/g\right)/\left[\sum \left(\bar{X}i-\bar{X}\right)2/\left(g-1\right)\right]$ was used to confirm the sample number. n indicates the sample number; g is the number of groups; $\bar{X}i$ and Si were the estimated means and standard deviations of every group. After setting the significant level (α) as 0.05 and type II error (β) as 0.1, the φ value was obtained according to φ value table. From the data we got from our previous studies and some other similar reports,^37^, ^38^ we confirmed the $\bar{X}i$ and Si. For in vivo observation of PDL regeneration under coupling of occlusal loading and MGF, the sample numbers of rats needed for validating each parameter in our study was determined 6 for immunofluorescence staining. Experimental rats were numbered and randomly sorted into each of the experimental animal groups using Excel. After extraction, all teeth were cleaned by rinses with physiological saline and kept dry on a metal tray at room temperature (25 ± 1°C) for 1 h. Before replantation, the root surfaces of the extracted teeth and the alveolar sockets were cleaned with physiological saline and sterile gauze to remove the tissues and the blood clots. The wall of the socket was never scraped to avoid further injury to the microenvironment of replantation. After replantation, the teeth were fixed with absorbable 11–0 thread to prevent teeth loss and displacement.

Following the random number table, the rats were randomly divided into four groups according to the adjuvant graft that was used. For the block food group (BF group), jelly was fed within 1 w after tooth avulsion and replantation, and then AIN‐76A block feed was provided until the samples were harvested. For the powder food group (PF group), jelly was fed within 1 w after tooth avulsion and replantation, and then AIN‐76A powder feed was provided until the rats were sacrificed. For the block food plus MGF treatment group (BF + MGF group), a collagen sponge containing 1000 ng/mL Goldspink MGF was added during tooth replantation, jelly was provided within 1 week after replantation, and then AIN‐76A block feed was provided until the samples were harvested. For the powder food plus MGF treatment group (PF + MGF group), a collagen sponge containing 1000 ng/mL Goldspink MGF was added during tooth replantation. Jelly was provided within 1 week after replantation, and then AIN‐76A powder feed was provided until the end of the observation period (Figure 1a). All rats were returned to their home cages, where they were observed for signs of pain, infection, and daily activity. All animal procedures performed in this study were reviewed and approved by the Animal Experimental Ethical Inspection of Fourth Military Medical University (No. Kq‐016) and were performed by the guidelines of the International Association for the Study of Pain.

Next, animals were sacrificed at 8 weeks after replantation to collect maxilla blocks. The maxillary block of the rats was removed following perfusion fixation with 4% paraformaldehyde in phosphate buffer. Micro‐CT was performed at a tube current of 50 μA and a tube voltage of 90 kV. The interlayer spacing was 20 μm. All data were recorded using an in vivo x‐ray micro‐CT system (Y. XLON, Germany). 3D visualization was achieved using VGStudio MAX 2.2 software (Ratoc System Engineering Co, Japan). The mandibles were reconstructed using the same software program to evaluate the cross‐sectional and sagittal views of the incisor teeth and their periodontium, as well as their surrounding bone. From the micro‐CT scanning images of the root of replanted teeth, we calculated the percentages of root resorption (Figures 6b and S6).

The histological analysis was performed as described previously. The tissue slices were observed and photographed with an optical microscope according to the method reported by Andreasen.^38^ The healing mode of PDL after dental trauma was divided into two categories: physiological periodontium healing and pathological healing. The latter involves three bone absorption types: surface absorption, alternative absorption, and inflammatory absorption. The following eight observation sites on tooth root cross‐sections were used: middle‐buccal point, middle‐lingual point, mesial point, distal point, mesial‐buccal proximal point, distal‐buccal proximal point, mesial‐lingual proximal point, and distal‐lingual proximal point. Referring to the periodontal histology score scale, the healing effect on the replanted teeth in each group was evaluated, and the types and incidence of root resorption were analyzed. The number of teeth with root resorption and the percentage of resorption points relative to the total points of each group (presented as the mean ± standard error, x% ± SD) were calculated and statistically analyzed and following the methods we have reported previously.^37^ The collagen in PDL was measured with Masson trichrome staining as area of blue pixels.

### 2.8 Statistical analyses

All in vitro experiments were repeated at least three times. The results are reported as the means ± standard deviations and were compared using a one‐way analysis of variance (ANOVA) combined with the Student–Newman–Keuls post hoc test. For the in vivo results, the percentage of each histological classification for each root and each testing group was calculated. The differences among groups were statistically compared using the Kruskal–Wallis H test and were further analyzed using the Mann–Whitney *U* test. A *p*‐value <0.05 was accepted as statistically significant. The analyses were performed using SPSS 19.0 software (SPSS, USA).

## 3 RESULTS

### 3.1 Occlusal loading maintains PDL morphological features and promotes MGF expression in the PDL in a rat model

To test the effects of occlusal forces on PDL, we established a rat model to assess the relationship between occlusal loading and PDL regeneration with powder/block food feeding for bite force adjustment (Figure 1a). We observed that the body weights of rats increase normally over time in both the block food group (BF group) mimicking “normal occlusal force” and powder food group (PF group) mimicking “reduced occlusal force” (Figure 1b). Except for the way the rats ate, the food intake of the two groups kept basically same by evaluating of the total consumption amount of two kinds of diets in different groups. The scanning site of tooth root area was selected at 1 mm close to the internal tooth margin for reconstruction (Figure 1c). One hundred and fifty scanned pictures were selected to establish the periodontium model (Figure 1d). Micro‐CT scanning of both BF group and PF group maxillary were performed. Horizontal sections and coronal sections of Micro‐CT scan highlighted upper central incisors and PDL structures. 3D reconstruction of PDL of the maxillary central incisors of rats were obtained using micro‐CT. (Figure 1e), we calculated the average thickness of the periodontium. The average thickness of the periodontium in the BF group remains stable at 0.14 ± 0.02 mm at week 2, week 4, and week 8, while that of the PF group decreases gradually with prolonged feeding time (0.13 ± 0.01 mm at week 2, 0.12 ± 0.02 mm at week 4, and 0.11 ± 0.01 mm at week 8). There exists a significant difference between the two groups at week 8 (*p* < 0.05; Figure 1f).

Next, we checked the morphological features of PDL using hematoxylin–eosin (HE) and Masson's trichrome staining (Figure 1g–i). At week 8, PDL cell density decreases in the PF group (*p* < 0.05; Figure 1g,h). And PDL fibers of the BF group are arranged neatly and perpendicular to the cementum, while PDL fibers are irregularly arranged (Figure 1i). The bone mineral density (BMD) significantly decreases in the PF group (*p* < 0.05; Figure S1). We also checked the MGF expression in the periodontal ligament of both the BF and PF groups at week 8 through immunohistochemical staining. Occlusal loading leads to an increase in MGF expression in the PDL (Figure 1j), while MGF expression is significantly higher in the BF group than PF group as reflected by the average optical density (AOD) of immunohistochemical staining (*p* < 0.05; Figure 1k). Further, high throughput sequencing results from gene expression omnibus (GEO) Datasets (accession number: GSE112122) revealed that there are higher expressions of Scleraxis in PDLSCs subjected to intermittent compressive force compared to control cells (Figure 1l).^39^

### 3.2 Hydraulic pressure and MGF promote the differentiation of PDLSCs toward fibroblasts

During mastication, occlusal loading is the main mechanical stimulus that the PDL experiences. To assess the PDL responses to occlusal force in vitro, we mimicked the mechanical microenvironment using the multifunctional hydraulic cellular pressure to study the regenerative ability of PDLSCs (Figure 2a). As a highly vascularized tissue enriched with capillaries, PDL endures consistent stress and increased blood pressure during occlusal loading.^40^ During occlusal loading, increased blood pressure (hydraulic pressure) in PDL was correlated with the locations of the root resorption.^41^ Thus, hydraulic pressure was adopted as the main mechanical stimulation in this study. We investigated the effects of hydraulic pressure, MGF, or dynamic pressure combined with MGF on PDLSCs and the underlying mechanisms (Figures 2b and S3). We observed that MGF (both h‐MGF and G‐MGF), pressure, and pressure + MGF (both h‐MGF and G‐MGF) increase the Scleraxis protein levels (*p* < 0.05; Figure 2c). Notably, Scleraxis levels in the pressure + MGF (both h‐MGF and G‐MGF) are significantly higher than the pressure group. The protein levels of the osteogenic marker Osterix in the treatment group are not significantly altered compared with the control group (*p* > 0.05; Figure 2d).

The fluorescence staining showed that the fluorescence intensities of Scleraxis in the MGF (both h‐MGF and G‐MGF), pressure, and pressure + MGF (both h‐MGF and G‐MGF) treatment groups are stronger than those in the control group, whereas the fluorescence intensity of Osterix in the treatment groups remains unchanged. We also examined the patterns of actin filaments in PDLSCs that are sensitive to pressure. F‐actin fluorescence staining for cells in the pressure and pressure + MGF (both h‐MGF and G‐MGF) treatment groups is much more intense than control (Figure 2e). Scleraxis and Osterix positive cells were counted and the ratio of positive cells was calculated. It showed that the ratio of Scleraxis positive cells in the MGF (both h‐MGF and G‐MGF), pressure, and pressure + MGF (both h‐MGF and G‐MGF) treatment groups were significantly higher than that of the control group (*p* < 0.05; Figure 2f). In contrast, there was no significant difference in Osterix positive cell ratio among all the groups (Figure 2g).

### 3.3 MGF enhances the mechanobiological response in PDLSCs via Fyn/FAK signaling

**FIGURE 3:**
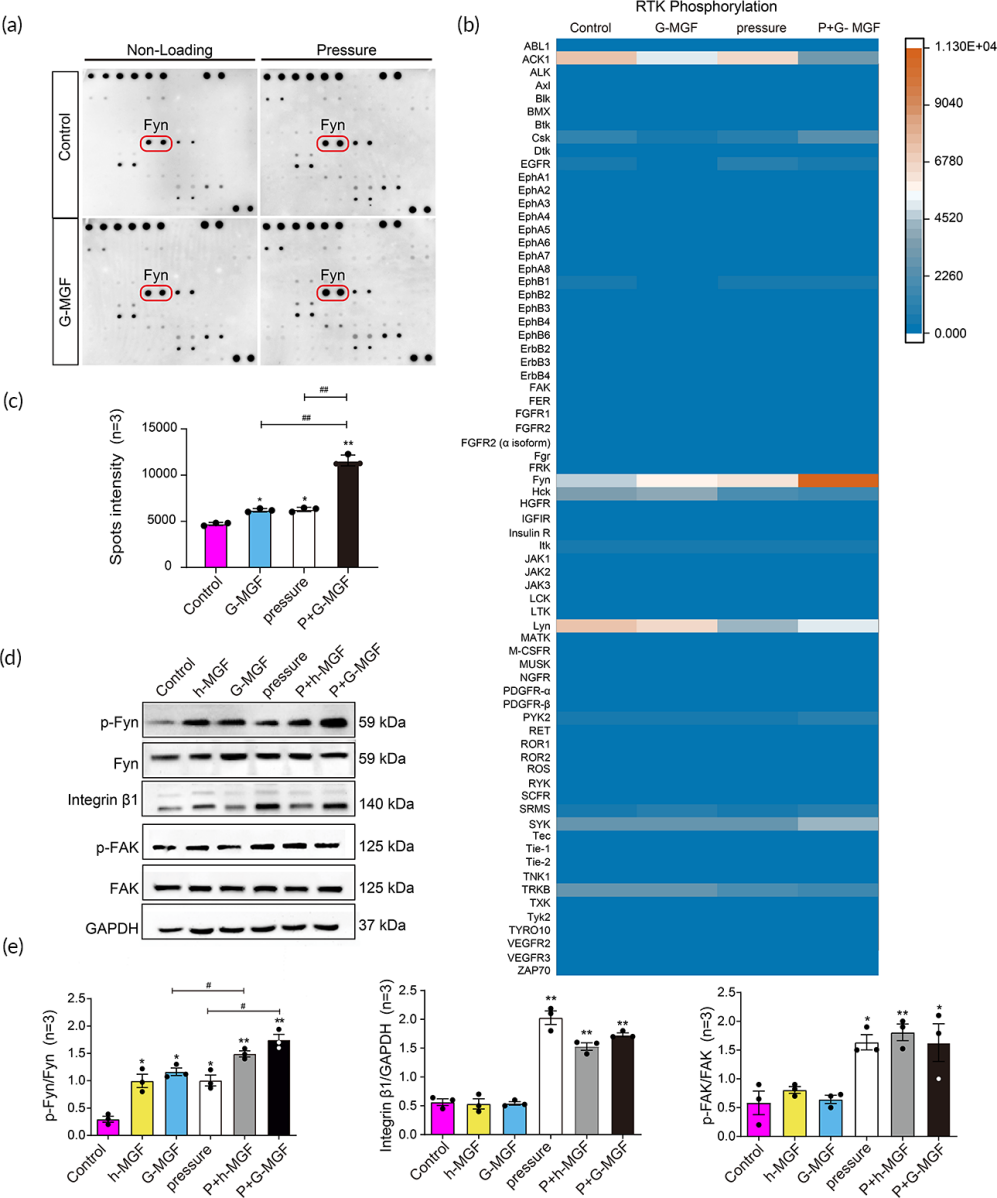
Non‐receptor type tyrosine kinases Fyn is the primary signaling pathway downstream of MGF. (a) Human RTK Phosphorylation Antibody Arrays were used to detect the phosphorylation levels of downstream proteins. (b) Heatmap analysis of phosphorylated protein levels. (c) Spot intensity of phospho‐Fyn. (d) Western blotting was conducted to detect the levels of Fyn, p‐Fyn, integrin β1, FAK, and p‐FAK. (e) Analysis of the differences in OD values for p‐Fyn, integrin β1, and p‐FAK (*p < 0.05, **p < 0.01, vs. control group; ^#^ *p* < 0.05, ^##^ *p* < 0.01, vs. G‐MGF or pressure group, *n* = 3).

To understand the mechanism of how hydraulic pressure and/or MGF influence PDLSCs, we checked the phosphorylation levels of tyrosine kinase signaling pathways in PDLSCs and screened the downstream receptor of the mechano‐growth factor using a human tyrosine kinase phosphorylation antibody chip (Figures 3a, S4 and S5). The phosphorylation levels of various kinds of growth factor receptors (e.g., EGFR, FGFR1/2, HGFR, IGF‐1R, insulin R, PDGFR‐α/β, and VEGFR2/3) remain unchanged, while the levels of phospho‐Fyn from the Src family of nonreceptor tyrosine kinases exhibit the greatest increase among the tyrosine kinases (Figure 3b). Either pressure or MGF stimulation elevates the levels of phospho‐Fyn, while their combination results in an even higher phosphorylation level of Fyn compared to those in other groups (*p* < 0.01; Figure 3c).

Besides, we also detected the protein levels of Fyn and other mechano‐transduction molecules including integrin and FAK. The expressions of phospho‐Fyn are significantly higher in MGF (both h‐MGF and G‐MGF) and pressure + MGF (both h‐MGF and G‐MGF) treatment groups. The protein level of phosphorylated integrin β1 and FAK in pressure and pressure + MGF (both h‐MGF and G‐MGF) treatment groups are significantly higher compared with those in control group and MGF (both h‐MGF and G‐MGF) treatment group (*p* < 0.05). However, MGF stimulation alone does not upregulate integrin β1 or FAK phosphorylation (Figure 3d,e).

**FIGURE 4:**
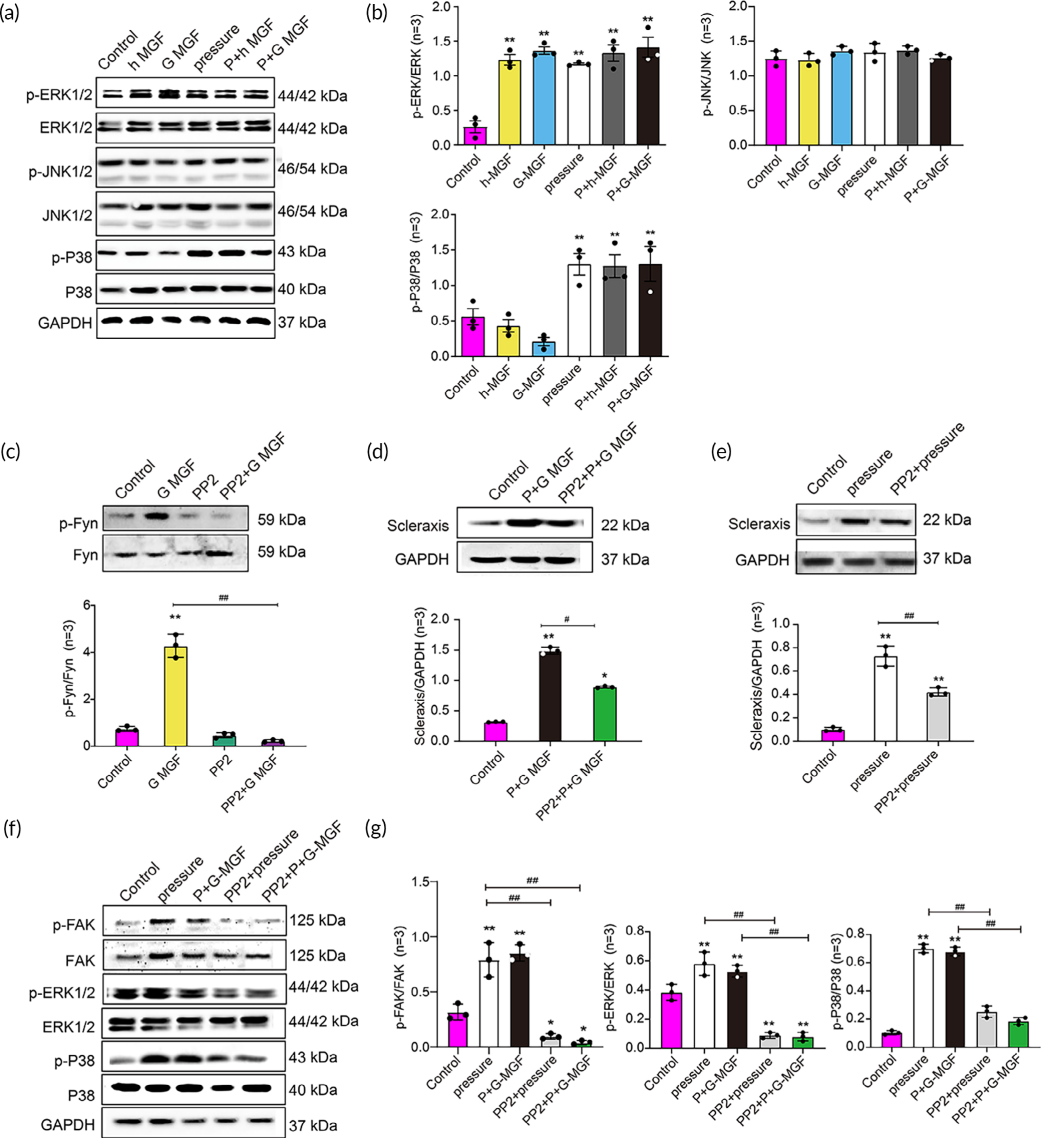
Fyn‐FAK and MAPK signaling mediates the mechanochemical coupling of MGF and mechanical pressure. (a, b) The levels of ERK1/2, p‐ERK1/2 (Thr202/Tyr204), JNK1/2, p‐JNK1/2 (Thr183/Tyr185), p38 and phospho‐p38 (Thr108/Tyr182) were detected using Western blotting. (c) The levels of Fyn phosphorylation were detected using Western blotting (**p < 0.01, compared with the control group; ^##^ *p* < 0.01, compared with the G‐MGF group, *n* = 3). (d, e) The levels of Scleraxis were detected using Western blotting. (f, g) Phospho‐FAK Tyr397, p‐ERK1/2 (Thr202/Tyr204) and phospho‐p38 (Thr108/Tyr182) protein levels after treatment with a Fyn inhibitor (PP2) (*p < 0.05, **p < 0.01, compared with the control group; ^#^ *p* < 0.05, ^##^ *p* < 0.01, compared with the pressure or P + G‐MGF group, *n* = 3).

Furthermore, the expressions of MAPK signaling pathway proteins including ERK1/2, phosphor‐ERK1/2 (Thr202/Tyr204), JNK1/2, phosphor‐JNK1/2 (Thr183/Tyr185), p38, and phosphor‐p38 (Thr180/Tyr182) in PDLSCs exposed to pressure and MGF were detected using a MAPK family phosphorylation antibody kit. After MGF (both h‐MGF and G‐MGF), pressure, and pressure + MGF (both h‐MGF and G‐MGF) treatments, the level of ERK1/2 phosphorylated at Thr202/Tyr204 was significantly higher than the control group (*p* < 0.05). The level of the p38 protein phosphorylated at Thr108/Tyr182 was significantly higher in the pressure and pressure + MGF (both h‐MGF and G‐MGF) treatment groups than that of the control group (*p* < 0.05). However, the level of phospho‐JNK1/2 shows no variation in all groups (Figure 4a,b).

There was a dramatic increase in the level of phosphor‐Fyn under the treatment of MGF (*p* < 0.05), which could be significantly reversed by the PP2 (Src inhibitor; Figure 4c). Then we checked the role of Fyn in fibroblast differentiation of PDLSCs subjected to the combined treatment of P + G‐MGF using PP2. The fibroblast differentiation marker Scleraxis increased significantly in the P + G‐MGF group, whereas adding PP2 reversed the elevated protein expression tendency, suggesting that the upregulation of Scleraxis was largely Fyn‐dependent (*p* < 0.05; Figure 4d). Likewise, Scleraxis expression was remarkably up‐regulated under pressure. This effect could also be reversed by the addition of Fyn inhibitor (Figure 4e). Further analysis showed that down‐regulation of Fyn kinase activity leads to a significant decrease in the level of phospho‐FAK, phospho‐ERK1/2, and phospho‐p38 (Figure 4f,g).

### 3.4 MGF and pressure enhances the expression of Scleraxis in PDLSCs by mathematical modeling analysis

**FIGURE 5:**
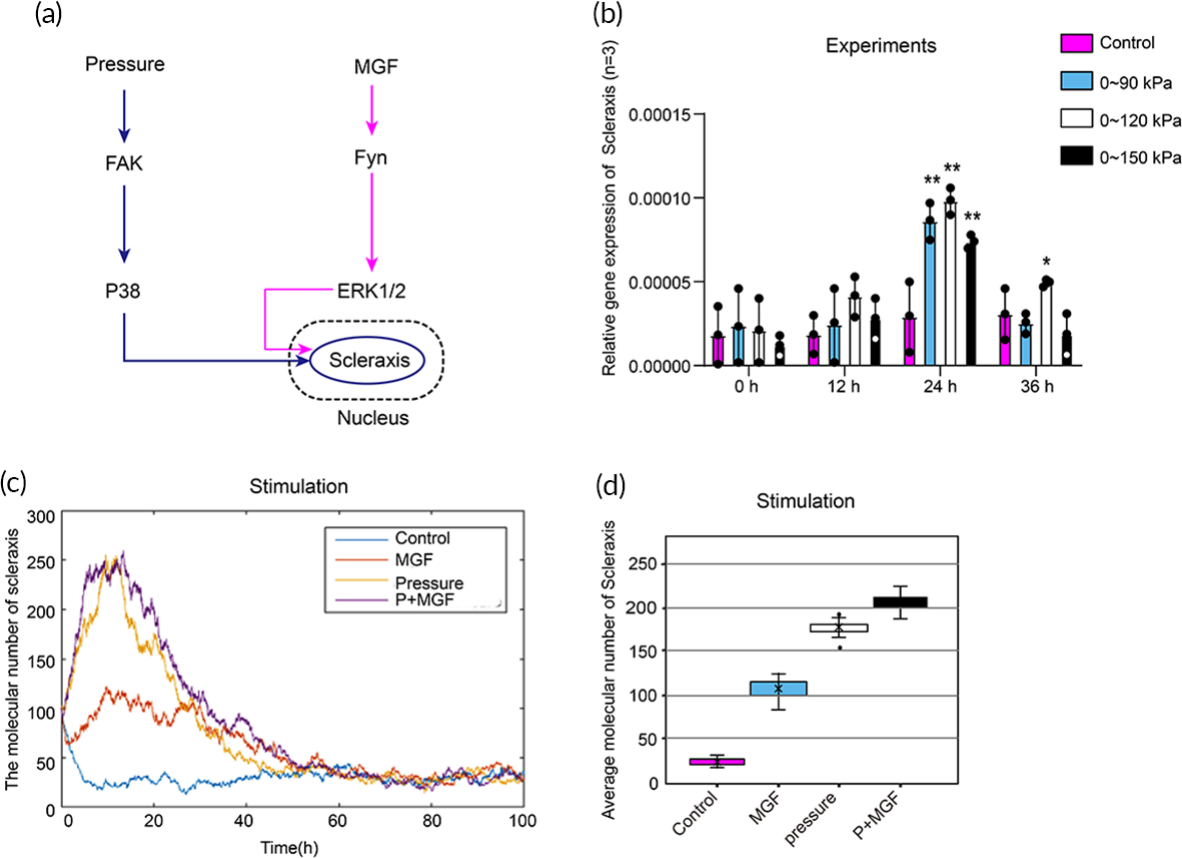
Mathematical modeling analysis the regulation of Scleraxis by MGF and pressure. (a) The mathematical model for the critical downstream pathways regulated by MGF and pressure. (b) Real‐time PCR showed that the MGF mRNA of PDLSCs could be significantly up‐regulated by the hydraulic pressure (*n* = 3 experiments). (*p < 0.05, **p < 0.01, compared with the control group, *n* = 3). (c) Average molecular number of Scleraxis between 12 and 24 h. (d) The mathematical model simulation results showed that MGF, pressure, and pressure + MGF regulate the expression of Scleraxis.

To further study the roles of MGF and pressure in the regulation of PDLSCs differentiation to fibroblasts, we performed experimental observations and also developed a mathematical model to analyze the mechanism of mechanical reinforcement in our previous experiments. The model mainly captured the three‐potential regulation between molecules observed experimentally: (1) Pressure mediated the activation of FAKY397; (2) FAK‐p38 axis mediated the expression of Scleraxis and (3) MGF‐Fyn‐ERK axis promotes the expression of Scleraxis (Figure 5a). After treatment with hydraulic pressures of 0–90, 0–120, and 0–150 kPa for 0, 12, 24, and 36 h, the expression of MGF mRNA is significantly increased at 24 h, especially in the 0–120 kPa group (*p* < 0.05; Figure 5b). Later, all rate constants in the model fit our experimental observations. Simulation results showed that the Scleraxis presents a maximum value at 12–24 h and decreases to a normal level at 36–40 h (Figure 5c). Besides, the simulation also suggested that the average molecular number of Scleraxis is upregulated under “MGF,” “pressure,” “pressure + MGF” (Figure 5d) which showed consistent with our experiment observations.

### 3.5 MGF promotes mechano‐dominated PDL regeneration in vivo

**FIGURE 6:**
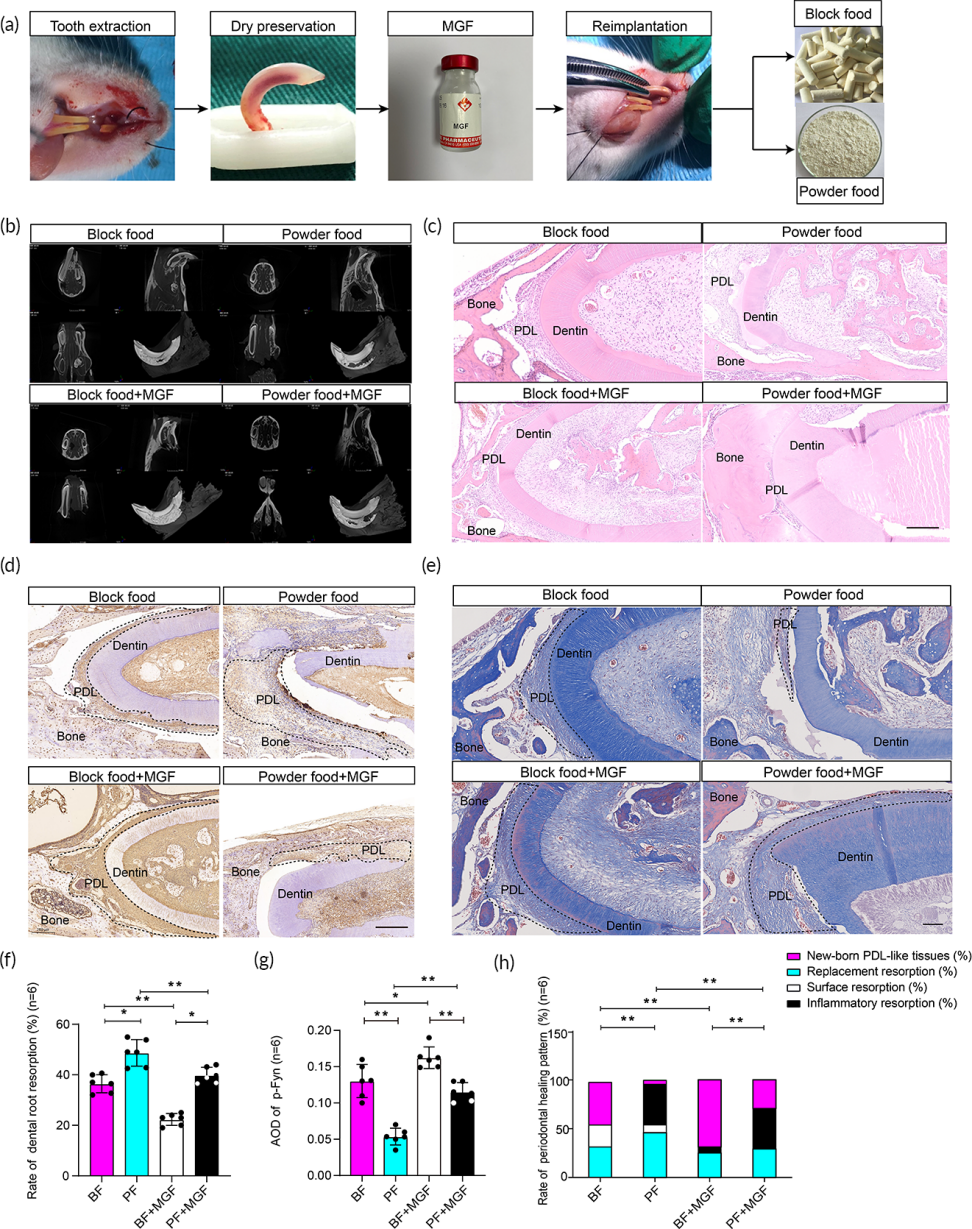
MGF greatly promotes PDL regeneration of the avulsed tooth after replantation in rats. (a) Schematic diagram of the tooth reimplantation strategy for avulsed teeth in rats. (b, f) Micro‐CT reconstruction images of rat upper incisors after 8 weeks of replantation. Root resorption craters with different forms and rough cementum areas were observed in the different treatment groups. 3D image of replanted incisors were reconstructed. The root absorption ratio of reimplanted incisors among the BF (block food) group, PF (powder food) group, BF + MGF (block food + MGF) group, and PD + MGF (powder food + MGF) group were compared. (c, h) HE staining images of maxillary central incisors from different groups and comparison of the newborn PDL‐like tissue ratio and absorption ratio. (d, g) Immunohistochemical examination of p‐Fyn expression and the statistical results of average optical density value of the p‐Fyn from diverse groups. (*p < 0.05, **p < 0.01, *n* = 6). (e) Collagen in PDL was stained with Masson's trichrome staining.

To evaluate the function of MGF and occlusal loading on periodontal ligament regulation, we established a rat model with delayed avulsed tooth replantation for PDL regeneration, and the animals were fed with powder or block food for bite force adjustment (Figure 6a). Micro‐CT reconstruction images of rat upper incisors after 8 weeks of replantation. Root resorption craters with different forms and rough cementum areas were observed in the different treatment groups (Figure 6b). 3D reconstructed replantation incisors volume and healthy side incisors volume, the root resorption percentage was calculated. The percentage of root resorption in the block food + MGF treatment group (BF + MGF group) is the lowest and is significantly lower than the other groups (*p* < 0.05). Notably, the percentage of root resorption in the powder food + MGF‐24E treatment group (PF + MGF‐group) is approximately equal to that in the BF group (*p* > 0.05; Figure 6f).

HE staining images of the avulsed and replanted teeth were histologically employed to evaluate the periodontal healing of replanted teeth. In the BF group and BF + MGF group, we observed a small amount of absorption lacunae on the root surface of the replanted teeth. However, the proportion of PDL‐like tissue accounts for the majority of the root surface. The collagen fibers in these two groups are generally arranged in an orderly manner, with nearly the same direction and dense structure. In contrast, in the PF and PF + MGF groups, lots of absorption lacunae were on the root surface of the replanted teeth, and a large number of inflammatory cells infiltrate near the resorption lacunae (Figure 6c,h).

Based on the analysis of HE staining images, we found that the proportion of PDL‐like tissue in the PF group is 4.16% ± 7.22%, significantly lower than the other groups (*p* < 0.05). On the contrary, the proportion of PDL‐like structures is 68.75% ± 8.83% in the BF+ MGF group, higher than the other groups (*p* < 0.05). Furthermore, there exist varying degrees of replacement resorption in each group, and the resorption of the PF group is the highest (45.83% ± 31.45%). In addition, the proportions of inflammatory resorption in the PF and PF + MGF groups are 41.67 %± 14.73% and 41.67% ± 2.94%, respectively, which are significantly higher than both the BF and BF + MGF groups (*p* < 0.05; Table 1).

**TABLE 1:** Histological evaluation of periodontal healing patterns in different groups.

Groups	Treatment	Newborn PDL‐like tissues (%)	Replacement resorption (%)	Surface resorption (%)	Inflammatory resorption (%)
		$\bar{x}$(%) ± SD, X*	$\bar{x}$(%) ± SD, X*	$\bar{x}$(%) ± SD, X*	$\bar{x}$(%) ± SD, X*
Group I (*n* = 6)	Block food group (BF group)	43.75 ± 8.83	31.25 ± 8.84	22.56 ± 17.67	0
Group II (*n* = 6)	Powder food group (PF group)	4.16 ± 7.22^b^	45.83 ± 31.45^b^	8.05 ± 7.22^b^	41.67 ± 14.73^b^
Group III (*n* = 6)	Block food + MGF group (BF + MGF group)	68.75 ± 8.83^b^	25 ± 0.01	0^b^	6.25 ± 4.42
Group IV (*n* = 6)	Powder food + MGF group (PF + MGF group)	29.17 ± 7.22^b^	29.17 ± 14.43^b^	0^b^	41.67 ± 2.94^b^

*Note*: *n* is the number of teeth examined in each group; $\bar{x}$(%) is the mean group pathology index; X* is the number of teeth exhibiting a particular type of periodontal change.

^a^ Statistical analysis was performed using the Kruskal–Wallis test and Mann–Whitney *U* test (*p* < 0.05); the percentage of each healing pattern was selected as the index, and the results are presented as the mean percentages ± standard deviations (SD).

^b^ *p* < 0.05, compared with Group I (BF group).

From the immunohistochemistry staining of Fyn phosphorylation at Tyr397 in the PDL‐like tissue, we observed that occlusal loading and MGF lead to an increase in p‐ Fyn expression in the PDL as reflected by the average optical density (AOD) of immunohistochemical staining. p‐Fyn expression in the BF group is significantly lower than the PF group, while p‐Fyn expression in the BF + MGF group is significantly higher than the PF + MGF group (Figure 6d,g). Masson trichrome staining was performed on the PDL derived from the wound site, to assess the inflammatory response and collagen deposition during the healing process. In the PF (powder food) group and PD + MGF (powder food + MGF) group, the collagen fibers were disordered and arranged irregularly, along with an expansion of collagen deposition at 8 W. However, in BF (block food) group and BF + MGF (block food + MGF) group the collagen fibers arranged neatly (Figure 6e).

## 4 DISCUSSION

### 4.1 Biomechanical stimuli are essential for PDL functional maintenance and regeneration

As a dense connective tissue that connects the tooth root to the alveolar bone, PDL supports the teeth and conducts the bite force.^42^, ^43^ When a force is applied to the tooth in the process of biting, the tooth moves slightly in its socket, which inevitably induces stresses in the PDL. As the PDL is in a special mechanical microenvironment that connects two tissues with significantly higher stiffness,^44^ cells in the PDL will undergo a series of metabolic reactions after mechanical stimulation. Thus, the PDL can adapt to complex physiological functions and plays an important role in stress dispersion and buffering under occlusal loading, as well as the physiological movement of teeth under orthodontic force. Many studies have reported fiber system degradation^45^ and changes in growth factor^46^, ^47^ and other materials (e.g., NOS,^48^ chondroitin sulfate,^49^ decorin^50^) in the PDL in the absence of a masticatory load. The mechanoreceptors in the PDL of auto‐transplanted teeth might be impaired, making the teeth more susceptible to occlusal overloading, although the teeth appear to be in a relatively healthy condition.^51^ Clinical evidence has shown that two mechanical factors are necessary for the replanted tooth to obtain optimal periodontal healing: low‐magnitude strain induction to the healing tissues and a controlled micromovement of the tooth in the traumatized socket (~50 μm), which effectively prevents dentoalveolar ankylosis.^52^ To our knowledge, this study provides the first direct imaging and quantitative evidence that occlusal force deprivation leads to a decrease in the thickness of the periodontium, a reduction in periodontal bone quality, and an increase in the rate of pathological healing of the PDL after avulsion. All of the above‐mentioned works indicate that occlusal loading is closely related to PDL regeneration and functional maintenance, though the underlying molecular mechanism regulating these processes remains unclear.

### 4.2 MGF represents a promising treatment for promoting periodontium regeneration

MGF is an alternatively spliced isoform of IGF‐1, which is expressed in many tissues and encodes a unique E‐domain region that can regenerate tissues.^11^, ^12^, ^53^ PDLSCs, as a type of mesenchymal stem cells derived from the periodontal ligament, have tissue specificity and multidirectional differentiation capability,^54^, ^55^ rendering them with potential application value in periodontal tissue regeneration.^56^, ^57^ Since the present study focused on the regeneration and repair of periodontal membrane after injury, the MGF secretion and response to MGF in PDLSCs were emphatically observed. In the present study, we first reported that occlusal stimuli increase the expression of a specific mechanical stimulation‐related growth factor, MGF, in PDLSCs. Also, the function and molecular mechanism of MGF‐promoted differentiation of PDLSCs toward fibroblasts were revealed both in vitro and in vivo for the first time. It was reported that MGF exerts its function on muscle repair and regeneration after injury or training.^58^, ^59^ The present results manifested that MGF could also serve as an important treatment to promote PDL regeneration under occlusal pressure, which thus might be a promising tool to improve the treatment of periodontal defects.

There were two kinds of MGF‐24E commercially available at present, human MGF (h‐MGF) and Goldspink MGF (G‐MGF). Both of them were chosen for the present study. Generally, these two MGF are chemically synthesized peptides and share high similarity. The h‐MGF harbors exactly the same amino acid sequence to human being. The G‐MGF shares identical amino acid sequence to h‐MGF except for the substitution of arginine at site 23 (Arg23) with histidine (His), but this substitution may not affect the function of MGF, for the amino acid at site 23 in diverse kinds of animals was not conserved. More importantly, the amino acid of MGF at site 23 in majority of animals is histidine. In addition, the arginine of G‐MGF at sites 14 and 15 are D‐arginine, while the arginine in h‐MGF peptide at the same sites were natural L‐arginine, this replacement may harbor the potential to increase MGF stability. The results demonstrated that both h‐MGF and G‐MGF showed similar cellular and molecular effects on PDL.

This study might provide new strategies and ideas for patients with dental trauma of avulsion. Our in vivo study revealed that MGF promotes healing of the PDL after delayed replantation of avulsed teeth in rats. However, even after the administration of an additional dose of MGF, the proportion of PDL‐like tissue in occlusal loading‐deprived rats is still lower than normal occlusion groups, suggesting that MGF still does not completely replace the bite force. The potential roles of MGF and masticatory load in periodontal ligament repair and regeneration are worth further study.

### 4.3 MGF enhances the mechanobiology effects through Fyn signaling

MGF following mechanical stimulation enhances the effect of pressure on promoting fibroblast differentiation, suggesting that MGF must have interacted with the mechano‐transduction pathway at a certain point. What signaling pathways regulate the additive mechanical effect of MGF? Many previous studies have shown that MGF does not depend on binding to IGF‐1R to exert its function and blocking the IGF‐1R do not result in the function loss of MGF.^13^, ^60^ These implied that although MGF stimulation has no effect on IGF‐1R activation and protein expression, it promotes S6 ribosomal protein phosphorylation, which suggested the activation of protein synthesis.^61^ Currently, the receptor of MGF and the molecular mechanisms have not been completely elucidated. Since MGF is a splicing variant of IGF‐1, the present work initially aimed to identify the classic tyrosine kinase‐signaling pathway that generally interacts with growth factors. High‐throughput screening of phosphorylated tyrosine kinase antibody chips was employed in our preliminary experiment to detect the differential signaling proteins in PDLSCs exposed to pressure or MGF alone or in combination. A variety of growth factor receptors on the chip, including EGFR, FGFR1/2, HGFR, IGF‐1R, insulin R, PDGFR‐α/β, and VEGFR2/3, are not activated. A heatmap found that Fyn and SYK were up‐regulated compared with control group. Among these proteins, optical density value of the phospho‐Fyn was significantly elevated. And the optical density of phospho‐Fyn displayed the biggest increase. Therefore, we chose Fyn kinase as our research focus. The present study is the first to show that Fyn kinases activation may be the most critical molecular event in the additive mechanical effect of MGF.

### 4.4 Crosstalk between Fyn and FAK signaling contributes to the additive mechanical effect of MGF

**FIGURE 7:**
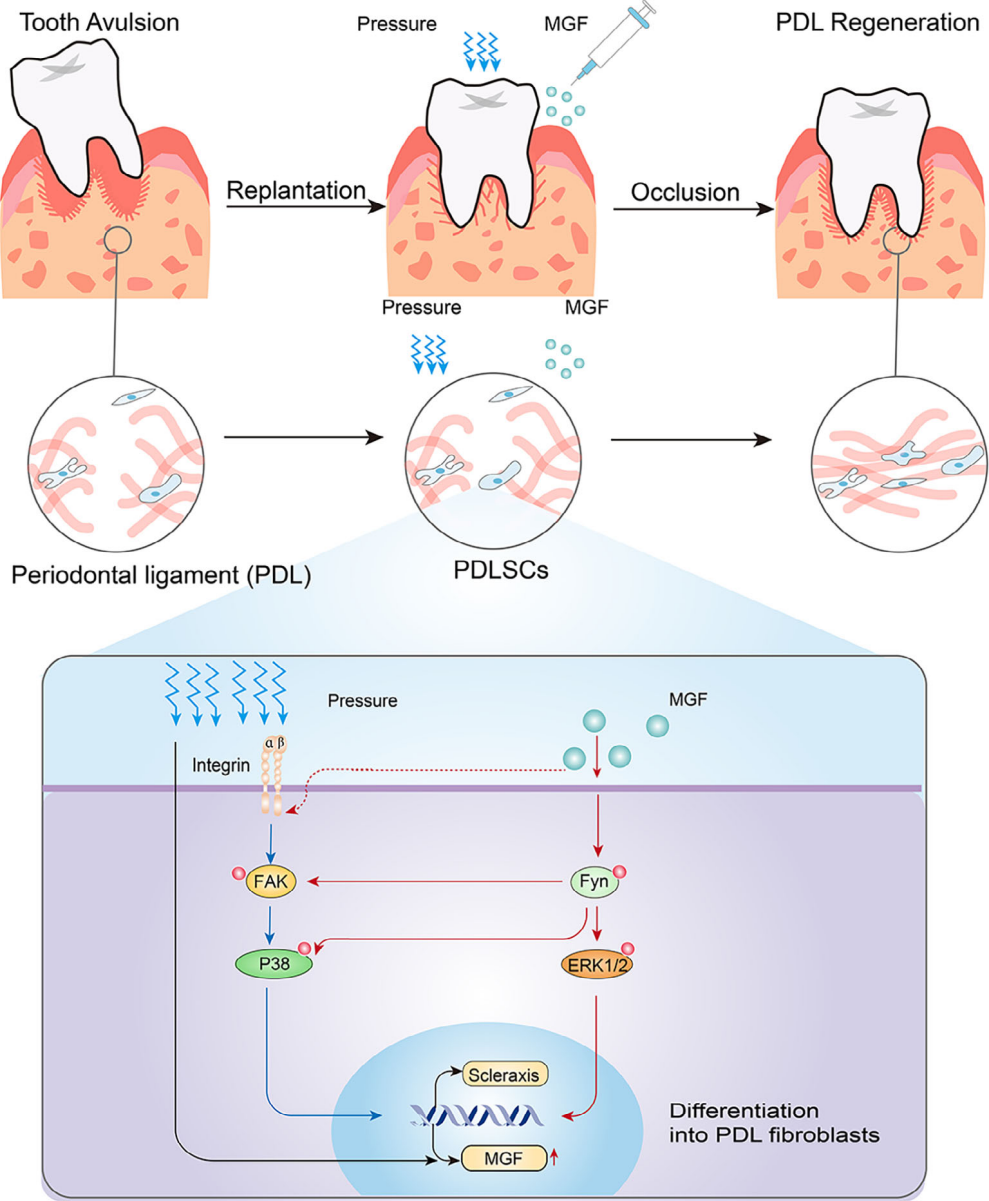
Schematic diagram of mechanochemical coupling of pressure and MGF for PDL regeneration. Tooth avulsion results in breakage of the PDL. The key to successful treatment is PDL regeneration after replantation. Mechanical pressure activates integrin β1, FAK, and p38 signaling, which may exert a wide range of positive biological effects on PDLSCs, including MGF expression and PDLSCs differentiation toward fibroblasts. Exogenous MGF enhances the expression of Scleraxis via Fyn‐FAK crosstalk and subsequent ERK1/2 and p38 phosphorylation. Moreover, Integrins might act as the potential receptor of exogenous MGF signaling and, in turn, transduce mechano‐enhancing Fyn‐FAK crosstalk and activation, through which the mechanical stimulation and MGF exert an additive facilitation on PDL regeneration.

How does Fyn kinases affect the mechanotransduction signal molecules after activation under MGF? We found that levels of Integrin β1 and the phosphorylation of Focal adhesion kinase (FAK) at Tyr397 in the P and P + MGF groups are increased significantly. The activation of Fyn and ERK, and the promotion of fibroblast differentiation, are most significantly increased by the combination of pressure and MGF. The Src inhibitor substantially reduces the differentiation‐promoting effect of the combined stimulation, suggesting that the synergistic effect of pressure and MGF on fibroblast differentiation is partially dependent on Src. Moreover, when Fyn is inhibited, increased phosphorylation of FAK at Tyr397 in cells under pressure is substantially reduced. Src family kinases bind to FAK phosphorylated at the Tyr397 residue, thereby stimulating FAK catalytic activity.^62^ In the present study, the Src inhibitor largely weakens the differentiation‐promoting effect of the combined stimulation, suggesting that the synergistic effect of pressure and MGF on fibroblast differentiation is partially dependent on Fyn. Fyn likely contributes to strengthening FAK activation during pressure‐induced fibroblast differentiation, which forms the key link to the additive mechanical effect of MGF. Further downstream, studies have indicated that MAPK plays an important role in regulating Scleraxis expression in cells.^63^, ^64^ In the present study, chemical stimulation with MGF alone activates ERK and mechanical stimulation alone activates both ERK and p38. Their co‐stimulation exerts a certain synergistic effect on activating MAPK signaling. The results confirmed the mechano‐sensitivity of the MAPK signaling pathway, which can be reinforced by MGF and thus constitutes the auxiliary link in the additive mechanical effect (Figure 7).

With regard to the relationship between the Fyn and Integrin. It was shown that Fyn resides in cholesterol‐rich microdomains, termed lipid rafts, on the plasma membrane.^65^ Lipid rafts are enriched with a variety of proteins, which is involved in multiple biological behaviors. Despite the lack of transmembrane proteins in lipid rafts, current study suggests that the proteins in lipid rafts play an important role in signal transduction which mediated by transmembrane protein.^66^ Besides, lipid raft also functions in growth factor receptors‐mediated integrin activation.^67^ Although the present results showed that exogenous MGF cannot affect integrin expression directly, inhibition of Fyn activities may suppress FAK activation under the stimulation of either pure MGF or the combination of both MGF and pressure. Since the FAK is a key downstream molecule of integrin, suggesting that inhibition of Fyn suppresses the activity of Integrin β1.^68^ Therefore, we speculated that integrin may be a potential receptor of MGF, which mediates the activation of Fyn in lipid rafts. During this process, integrin performs functions by altering its conformation and function, rather than the alteration of expression level. However, further investigations are still needed. Besides, pressure stimulation can be detected through many other mechanosensors in addition to integrins. It was found by the present work that MGF can exert a synergistic effect on FAK activation by membrane adhesion molecules under the mechanical stimulation. However, cells are bound to deform under pressure. Whether other mechanotransduction molecules such as piezo1^69^, ^70^ or nuclear lamina molecules such as linker of nucleoskeleton and cytoskeleton (LINC),^71^ which are involved in cell and nuclear membrane deformation, participate in the synergistic effect of pressure and MGF were worthy to be studied further.

## 5 CONCLUSIONS

To our knowledge, this study is the first to report the additive mechanical effect of MGF mediating mechanochemical coupling, which exerts a significant facilitation on PDL regeneration. The exciting discovery of the present study is that feasible pressure and MGF synergistically activate integrin β1, Fyn, FAK, and MAPK signaling, which may exert a wide range of positive biological effects on PDLSCs. MGF potentiates the effect of pressure at the molecular level of Fyn, which in turn transduces mechano‐enhancing signals by strengthening FAK activation. MGF exerts a significant effect on enhancing fibroblast differentiation when combined with mechanical stimulation. The results of the mathematical model showed that MGF could strengthen the mechanobiology in PDLSCs by an additive mechanical effect. Thus, we speculate that MGF may likely be a powerful cofactor that accompanies mechanical stimulation to strengthen the mechanobiological response. In summary, there is an additive mechanical effect of MGF mediated by Fyn/FAK crosstalk and subsequent ERK1/2 and p38 phosphorylation, which exerts a significant facilitation on MGF expression and PDLSC differentiation toward fibroblasts, and provides the basis for the in vivo result of enhanced PDL regeneration under the mechanochemical coupling of MGF and occlusal force.

## AUTHOR CONTRIBUTIONS

**Ying Zhao:** Data curation (equal); formal analysis (equal); methodology (equal); writing – original draft (equal). **Songbai Zhang:** Data curation (equal); formal analysis (equal); methodology (equal); writing – original draft (equal). **Bo Cheng:** Data curation (equal); formal analysis (equal); methodology (equal); writing – original draft (equal). **Fan Feng:** Data curation (equal); formal analysis (equal); methodology (equal); writing – original draft (equal). **Yue Zhu:** Data curation (equal); methodology (equal). **Yanli Liu:** Data curation (equal); formal analysis (equal). **Junjun Wang:** Data curation (equal); formal analysis (equal). **Dehui Zou:** Data curation (equal); formal analysis (equal). **Heng Ma:** Investigation (supporting); supervision (supporting); writing – review and editing (supporting). **Feng Xu:** Investigation (supporting); supervision (supporting); writing – review and editing (supporting). **Min Zhang:** Investigation (supporting); supervision (supporting); writing – review and editing (supporting).

## FUNDING INFORMATION

This work is supported by grants from the National Natural Science Foundation of China (31971248, 81901052), Shaanxi Science and Technology Innovation Team Project (2021TD‐46), and National Clinical Research Center for Oral Diseases Project of Military Stomatology (LCA202007).

## CONFLICT OF INTEREST STATEMENT

The authors have declared that no competing interest exists.

### PEER REVIEW

The peer review history for this article is available at https://www.webofscience.com/api/gateway/wos/peer-review/10.1002/btm2.10603.

## Supporting information


**Data S1:** Supporting Information.

## Data Availability

The data that support the findings of this study are available from the corresponding author upon reasonable request.
