# Supplementary material for: Mechanochemical coupling of MGF mediates periodontal regeneration
Source: Bioeng Transl Med. 2023 Oct 7;9(1):e10603. doi: 10.1002/btm2.10603 (PMC10771565; doi:10.1002/btm2.10603)
Supplement: Supplementary file 1 — Data S1: Supporting Information. [file BTM2-9-e10603-s001.docx]

Supplemental Information

**Mechanochemical Coupling of MGF Mediates Periodontal Regeneration**

Ying Zhao^1,2,#^, Songbai Zhang^1,#^, Bo Cheng^4,5#^, Fan Feng^1,#^, Teng Tu^1^, Jing Niu^1^, Dehui Zou^1^, Heng Ma^3,*^, Feng Xu^4,5,*^, Min Zhang^1,*^

1. Establishment and evaluation of occlusal force adjusted animal model

Occlusal force of rats was adjusted by given block food (AIN-76A, Moldiets, China), and powder food (AIN-76A, Moldiets, China) for 1, 2, 4, and 8 weeks. Micro-CT scanning of the rat’s mandibular first molar showed that with the extension of feeding time, the mineral density of the alveolar bone around the teeth increased in both block food and powder food groups (**Figure S1 a**). However, at the same time point, the amount of cancellous bone mass in the root bifurcation area of the mandibular first molar was higher in the block food group than in the powder food group. The bone volume fraction (BV/TV %) in the region of interest (ROI) area of the powder food group was significantly lower than that of the block food group at 8 W (*P*<0.01). And the bone surface acreage and volume ratio (BS/BV) in the ROI area in the powder food group were even significantly lower than that in the block food group at 4 W (*P*<0.01). The average trabecular bone thickness (TbTh) in the ROI region of the powder food group was significantly higher than that of the block food group at 4 W (*P*<0.01). Besides, at 4 W and 8 W, the average trabecular bone number (TbN) of the block food group were significantly higher than those of the powder food group (P<0.01) and the average trabecular bone spacing (Tb.Sp) was significantly larger in powder food groups than those of block food groups (P<0.01) (**Figure S1 b-f**).


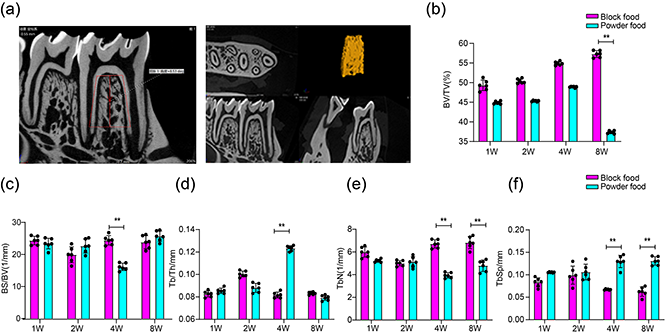


**Figure S1** Occlusal loading largely maintained the supportive tissue architecture maintenance of PDL. (a) Micro-CT scanning of the rats mandibular. VG Studio MAX 2.2 software was used to reconstruct the three-dimensional image to analyze the alveolar compartment of the rat mandibular first molar. (b) Bone volume fraction (BV/TV %) in the ROI area of the powder food group was significantly lower than that of the block food group at 8 W. (c) Bone surface acreage and volume ratio (BS/BV) in the ROI area in the powder food group was even significantly lower than that in the block food group at 4 W. (d) Trabecular bone thickness (TbTh) of the powder food group was significantly higher than that of the block food group at 4 W. (e) Trabecular bone number (TbN) of the block food group was significantly larger than those of the powder food group. (f) Trabecular bone spacing (Tb.Sp) was significantly larger in powder food groups than those of block food groups. (***P*< 0.01, *vs* the block food group, n = 6).

2. Isolation and purification of PDLSCs

PDLSCs were isolated and purified according to previously study^1^. Briefly, human impacted third molars were extracted from 3 systemically healthy adults (18-30 years of age) at the School of Stomatology, Fourth Military Medical University (FMMU). The teeth were immediately immersed into an ice-cold phosphate-buffered saline (PBS; Hyclone, Road Logan, UT, USA) solution that contained 100 U/mL penicillin/streptomycin (SigmaeAldrich, St. Louis, MO, USA) and transferred to the laboratory. The protocols were approved by the Institutional Research Review Board at the School of Stomatology of FMMU (No. IRB-REV-2022128) and that the experiments were performed in accordance with the Declaration of Helsinki (2008) for humans. The donors were fully informed and provided written consent for the donation of their teeth and for their subsequent use in this research project. PDL was gently separated from the intermediate 1/3 of the root surface and then digested in a solution of 3 mg/mL collagenase type I (Sigma-Aldrich, Germany) for 30 min at 37 ℃. After digestion, the tissue was centrifuged at 800 rpm for 5 min. Further, the supernatant was discarded, and the remaining cells and tissues were transerred into T-25 culture flasks containing 3 mL of α-minimum essential medium (DMEM, Sigma-Aldrich, Germany) with 15% fetal bovine serum (FBS, Every Green, China), 100 U/mL penicillin, and 100 g/mL streptomycin (Sigma-Aldrich, Germany), and then incubated at 37 ºC in 5% CO_2_. Medium was changed every 3 days. After reaching 80% confluence, the cells were passaged into new flasks.

STRO-1/MACS^+^ cells (Biolegend, USA) were separated by Magnetic-activated cell sorting (MACS). In summary, after the cells were detached from the flask with 0.25% trypsin. Single-cell suspensions (1×10^7^) were incubated with a primary STRO-1 IgM antibody (mouse anti-human 1:50) (Miltenyi Biotec, Germany) for 1 h at 4 ℃. After incubation, the cells were washed with phosphate buffered saline (PBS) containing 1% BSA for 3 times. Then the cells (1×10^7^) were resuspended with PBS containing 1% BSA and incubated with anti-Mouse IgM MicroBeads for 45 min at 4 ℃. STRO-1/MACS^+^ cells were isolated by magnetic separation with MS Columns according to the Instructions (Miltenyi Biotec, Germany). To evaluate the markers of PDLSCs, flow cytometry (Becton, Dickinson and Company, USA) was performed to assess the expression of various signature antigen markers. The cells were collected and supplemented in PBS with 3% BSA at 1× 10^6^ cells/mL. The single-cell suspensions were incubated with fluorescein isothiocynante (FITC) conjugated monoclonal antibodies for human CD90 (bioscience, USA), CD105, CD146, CD34, and CD45 (Invitrogen, USA) at 4 ℃ in dark. Non-immune immunoglobulin of the same isotype (bioscience, USA) was used as the negative control. The percentages of positively stained cells were analyzed with a flow cytometer (BD, USA) followed by the evaluation with FACSD via Version 6.1.3 software. To assess the colony forming efficiency, PDLSCs were cultured in 10 cm culture dishes with α-MEM (10% FBS) at a density of 1×10^3^ cells/well. After 14 days, cells were fixed with 4% formalin and stained with 0.1% crystal violet. Aggregates of more than 50 cells were scored as colonies.

To test the osteogenic differentiation potential of PDLSCs, cells were seeded onto 24-well plates (Corning, USA) at 1×10^4^ cells/cm^2^. After reaching 80% confluence, PDLSCs were cultured in osteogenic inductive medium (Cyagen Biosciences, USA) for 3 weeks. After osteogenic induction, the mineralization of PDLSCs was evaluated by Alizarin Red S (Cyagen Biosciences, USA) staining. To test the adipogenic differentiation potential of PDLSCs, cells were cultured on 6 well culture plates at a density of 1×10^4^ cells/cm^2^. When reaching 80% confluence, PDLSCs were cultured in adipogenic inductive medium (Cyagen Biosciences, USA) for 3 weeks. The intracellular lipid accumulation was evaluated with Oil red O (Cyagen Biosciences, USA) staining.

The PDL cells started to grow from the tissue at approximately day 4 to day 7 (**Figure S** **2a, b**). The cells exhibited a typical fibroblast like spindle morphology (**Figure S** **2c**). After magnetic-activated cell sorting (MACS), Stro-1^+^ cells were able to form clonogenic cell clusters (**Figure S 2d**). Flow cytometric analysis showed that Stro-1^+^cells were positive for CD73, CD90, CD105, CD146/ MUC18 and Stro-1, while negative for the surface markers CD14, CD34, and CD45(**Figure S 2e**). In osteogenic and adipogenic differentiation assays, mineralized deposits or lipid- laden vacuoles were observed in Alizarin red staining images or Oil Red O staining images after induction (**Figure S** **2f, g**).


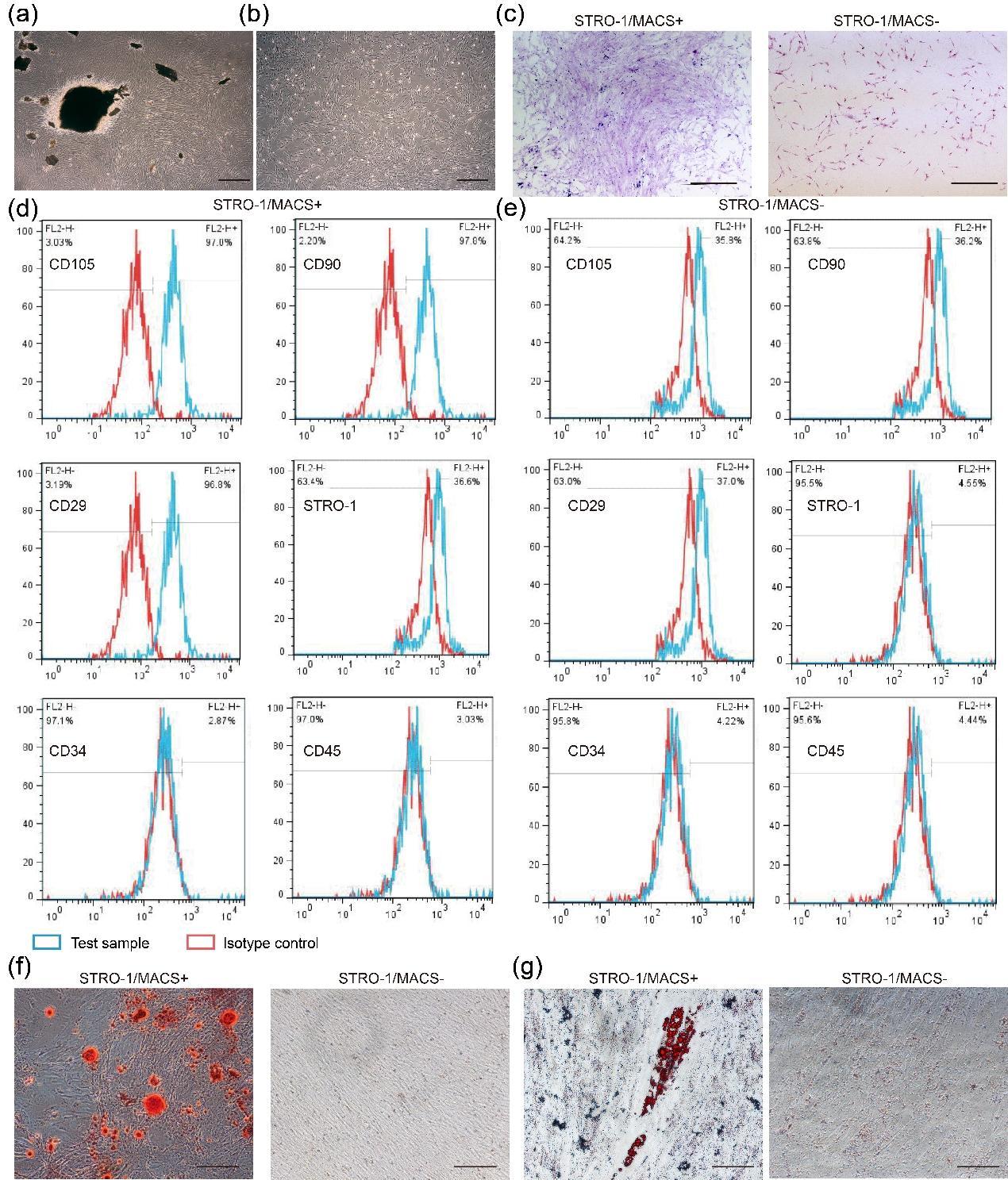


**Figure S2** Stro-1+ PDLSCs were purified from PDL cells by immune-magnetic bead sorting technology**.** (a) The cells started to grow from the PDL tissue at approximately day 4 to day 7. (b) The cells exhibited a typical fibroblast like spindle morphology. (c) After magnetic-activated cell sorting (MACS), Stro-1^+^ cells were able to form clonogenic cell clusters, whereas Stro-1^-^ cells did not. (d, e) Flow cytometric analysis showed that Stro-1^+^cells were positive for CD73, CD90, CD105, CD146/ MUC18, and Stro-1 and negative for the surface markers CD14, CD34, and CD45. The red and blue histograms represent isotype control and the tested cell sample respectively. (f) After the osteogenesis induction of Stro-1+ cells for 3 weeks, the nodules were positive for alizarin red staining. (g) In adipogenic differentiation assays, positive staining of Oil Red O in Stro-1+ cells were observed.

1. **The effect of hydraulic pressure and MGF on PDLSCs viability**

PDLSCs were cultured in dishes subjected to the periodic dynamic pressure exerted by the multifunctional hydraulic cellular pressure unit previously reported in our series of studies. Dobó-Nagy C et al. have shown that the ratio of apical pressure changes to the varying loading forces was 5.994 kPa N^-12^. The maximum bite force of the natural teeth of human healthy adults is between 300 and 600 Newtons (N)^3^. This calculation yielded an estimated apical pressure of 180~360 kPa, of which had no significant impact on cell bioactivity according to previous research^4^. Therefore, PDLSCs were treated with hydraulic pressures of 0-90 kPa, 0-120 kPa, and 0-150 kPa of 0.1 Hz for 1 h, and samples were taken at 0 h, 12 h, 24 h and 36 h after pressure. Then cells treated with 1 ng/mL, 10 ng/mL, 30ng/mL, 50 ng/mL, and 100 ng/mL human MGF (h-MGF) (Phoenix biotech, U.S.A.) and Goldspink MGF(G-MGF) (Phoenix biotech, U.S.A.). The amino acid sequences of h-MGF were Tyr - Gln - Pro - Pro - Ser - Thr - Asn - Lys - Asn - Thr - Lys - Ser - Gln - Arg - Arg - Lys - Gly - Ser - Thr - Phe - Glu - Glu - Arg – Lys. The amino acid sequences of G MGF were Tyr - Gln - Pro - Pro - Ser - Thr - Asn - Lys - Asn - Thr - Lys - Ser - Gln - D - Arg - D - Arg - Lys - Gly - Ser - Thr - Phe - Glu - Glu - His - Lys - NH2.

CCK8 results showed that the cell viabilities in the 0-120 kPa pressure group were significantly higher than those in the control groups at 0 h, 12 h, 24 h, and 36 h (**Figure** **S3a**). When cells were treated with human MGF (h-MGF) or Goldspink MGF (G-MGF) for 24 h, h-MGF or G-MGF of different concentrations can increase the viability of cells, the concentration of 100 ng/mL showed the highest viability (**Figure** **S3 b**).


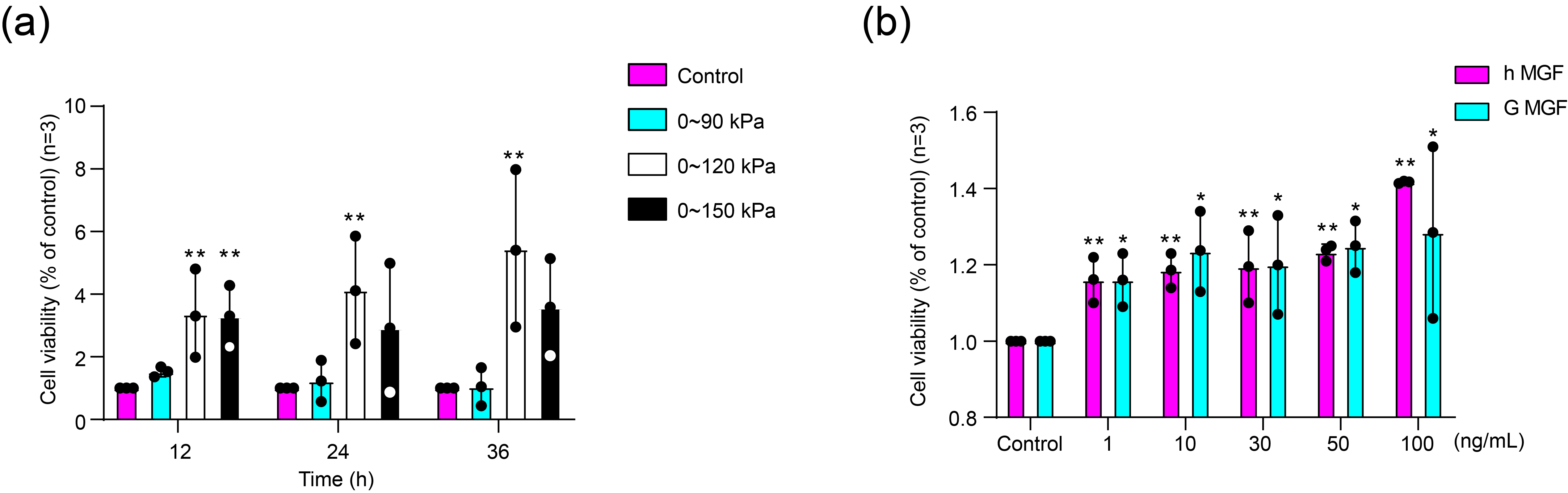


**Figure S3** The effect of hydraulic cellular pressure and MGF on PDLSCs viability. (a) Effects of the tested pressures on PDLSC viability (**P* <0.05, ***P* <0.01, compared with the control group). (b) Effect of h MGF and G-MGF on PDLSC viability (**P* <0.05, ***P* <0.01, compared with the control group).

1. **Phosphorylated protein analysis in cells stimulated by pressure and /or MGF using RTK phosphorylation antibody arrays**

The phosphor RTK-antibody array was purchased from Raybiotec Inc. Cells from different groups were lysed using lysis buffer (Raybiotec Inc, USA) supplemented with 1 mM phenylmethylsulphonyl fluoride. Lysates were analyzed by using the phosphor RTK-antibody microarray. The array consisted of 71 antibodies.


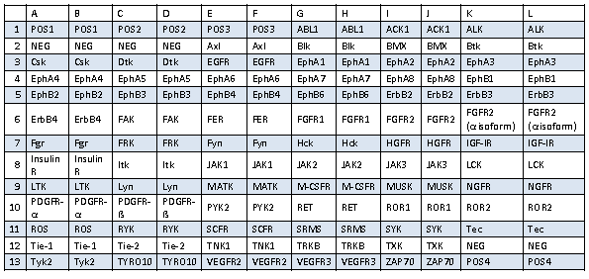


**Figure S4** The antibodys in Human RTK Phosphorylation Antibody Arrays. The array consisted of 71 phospho- RTK antibodies, which include receptor-type protein tyrosine phosphatase and non-receptor-type protein tyrosine phosphatase.

Except that mentioned above, the AKT phosphorylation pattern was also investigated. The results showed that MGF alone does not lead to the up-regulation of Akt phosphorylation, but the combination of pressure and MGF can result in the increase of Akt phosphorylation (**Figure S5**). These showed a similar pattern to p38. All the above results indicate that AKT is not an upstream signaling molecule of Fyn under MGF stimulation.


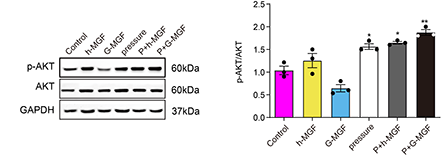


**Figure S5 Effects of MGF and pressure on Akt phosphorylation.** The levels of AKT, p-AKT were detected using Western blotting (**P*<0.05, ***P*<0.01, compared with the control group) (n = 3 experiments)

5. Methods for histological analysis of periodontal healing and root resorption

A grid with 8 radii was placed over the section of the rats’ root and oriented according to the labio-lingual and the mesio-distal axis of the tooth (**Figure S6 a**). The center of the grid was placed in the center of the root canal (**Figure S6 b**). Each grid line intersected withva root resorption area was registered. Root resorption was divided into surface resorption, inflammatory resorption, and replacement resorption according to definitions reported in an earlier report ^5^.


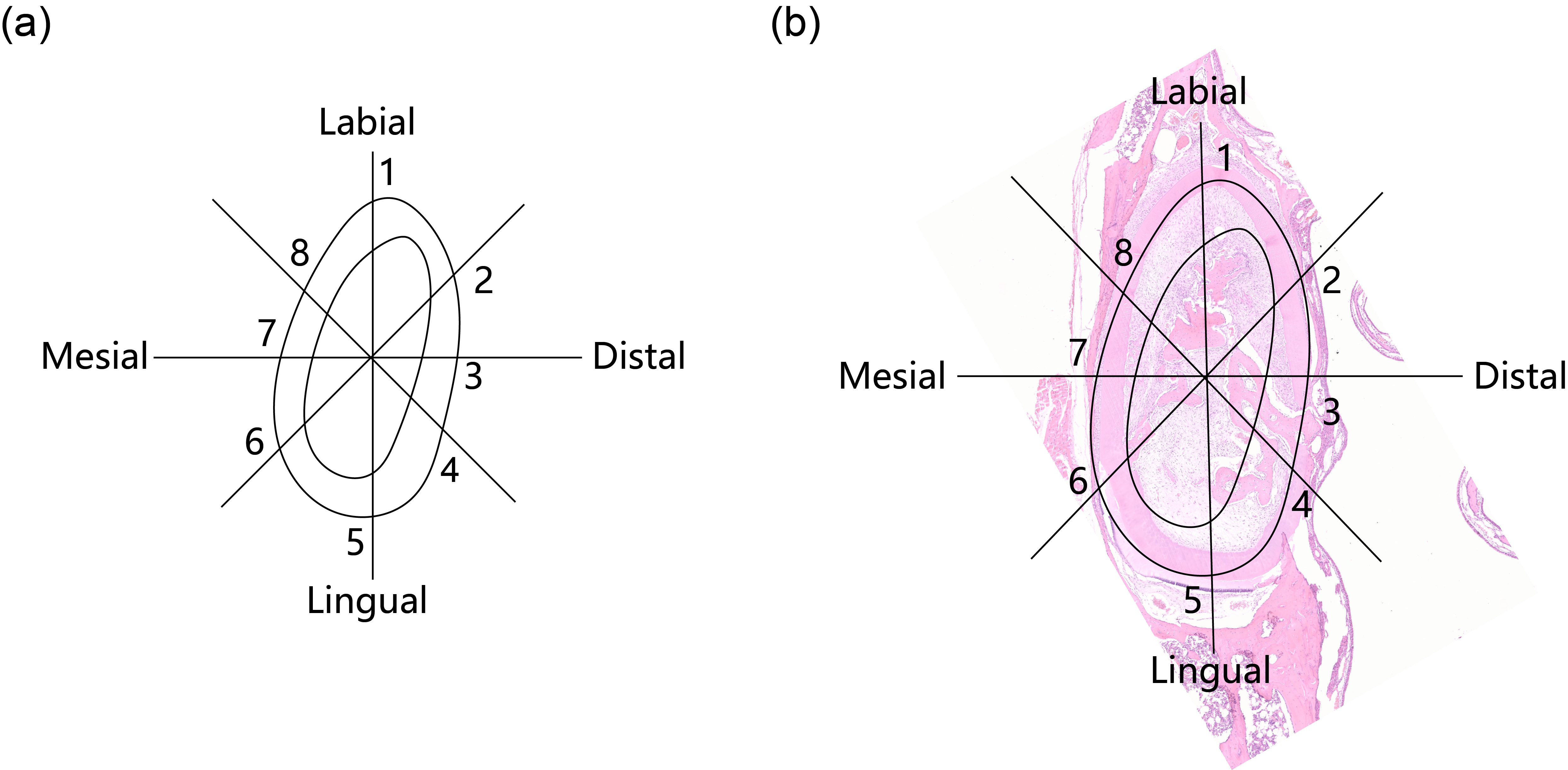


**Figure S6.** Methods employed for histological analysis of periodontal healing. (a) The tissue slices were observed and photographed with an optical microscope according to the method of Anderson^5^. Eight observation sites on the tooth root cross section were examined as the diagrammatic sketch indicated. (b) According to the references, the healing mode of PDL after dental trauma was divided into ideal periodontium healing and pathological healing. The latter involves three absorption types: surface absorption, alternative absorption, and inflammatory absorption. The exact healing type of each tooth at the 8 observation sites was recorded and analyzed. The pulp cavity was selected as the center, while the teeth were divided at a 45° angle as the regional unit.

1. Mathematical modeling analysis

Here, we developed a mathematical model to describe the additive effects of mechanical stimulation (pressure) and MGF on the expression of Scleraxis. The minimal dynamical model included three classes of molecules: FAK, MGF, and Scleraxis. The model captured the regulation relationship between molecules observed experimentally: (1) FAK-p38 mediated the expression of Scleraxis. Note that we assumed that the relationship between FAK Y397 level and mechanical stimuli, pressure here, is Hill function. Integrins are key molecules in such mechanosensing. Similar relationship between stiffness and FAK Y397 level is used in our previous model^6^; (2) MGF mediated the activation of FAK Y397 and (3) MGF-Fyn-FAK/p38 axis promotes the expression of Scleraxis. Here, we used the stochastic simulation to model the pressure and MGF mediated mechanical regulation in cells. The involved biochemical reactions about the above dynamical process are shown in Table S1.

**Table S1 Biochemical reactions about the mathematical modeling**


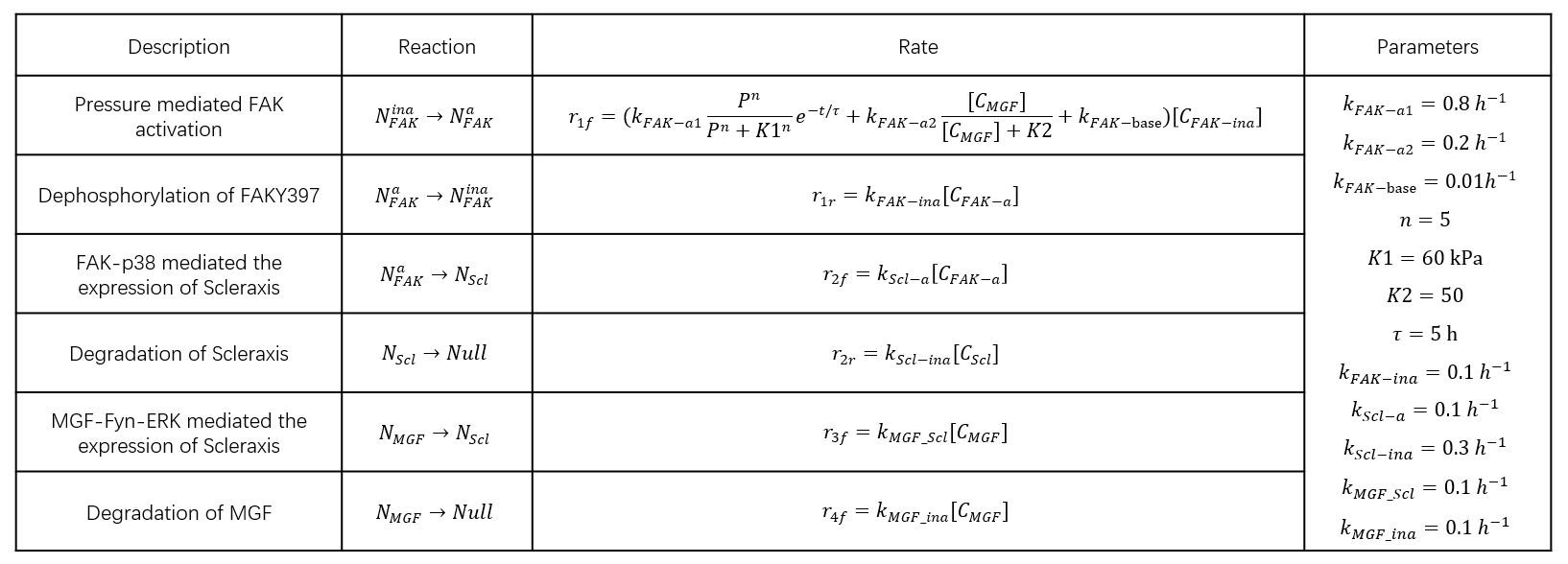


and are the activation rates of FAK by pressure and Fyn; is the base rate for FAK activation; is the deactivation rate of FAK; and are generation and degradation rates of Scleraxis mediated by FAK-p38 axis; is the generation rate mediated by MGF; P is the pressure; represents inactive FAK; represents active FAK; n, K1 and K2 are coefficients in Hill function. The [CFAK-ina], [CFAK-a], [CMGF], and [CScl] represent the simulated molecular number of inactivated FAK, FAK Y397, MGF, and Scleraxis in single cell.


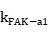

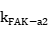

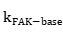

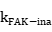

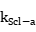

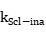

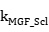

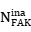

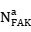


Here, for simplify, molecular number of FAK proteins is set 1000, the same order of magnitude as Rho GEF ref. However, due to the lack of experimental evidence of relevant rate parameters, we set all rate constants are 0.1 s^-1^. Actually, the main conclusions drawn from the model were insensitive to the values chosen for rate constants or initial molecular number or rate constant. Models were solved numerically using Matlab (The Mathworks, Natick, Massachusetts).

From the results of simulation, we can get the expression trend of FAK Y397 (**Figure 5**) and Scleraxis (**Figure 5**) with time. And we get the average value by running 20 simulation calculations. Consistent with the experimental results, the expression of Scleraxis under the cumulative effect of pressure and MGF tends to increase, that is, there is a cumulative effect (**Figure 5**).

1. MATLAB code of mathematical model

Matlab codes are as follows:

clear

clc

% R1: FAKina->FAKa

% R2: FAKa->FAKina

% R3: FAKa->Scl

% R4: Scl->null

% R5: MGF->Scl

% R6: MGF->null

F_Arry = zeros(100,1);

for j = 1:20

%======

% Parameters

t = 0; % Simulation time

P = 120; % P = 0 kPa or 90 kPa or 120 kPa or 150 kPa;

C_Scl = 100; % Molecular number of Scl := 0

C_MGF = 100; % Molecular number of MGF := 0 or 100

C_FAKina = 1000; % Molecular number of activated FAKa := 1000

C_FAKa = 0; % Molecular number of inactivated FAKa := 1000

K1 = 60; % Hill function coefficient

K2 = 50; % Hill function coefficient

Ncyc = 30000;

m = 1;

total = 0;

TimeArry = zeros(Ncyc,1);

C_R_Arry = zeros(Ncyc,1);

C_M_Arry = zeros(Ncyc,1);

C_FAK_Arry = zeros(Ncyc,1);

for i = 1:Ncyc

%======

% Rates of reactions

r1f = 0.8 * P^5 / (P^5 + K1^5) * (exp(-t/5)) + 0.1 * C_MGF / (C_MGF + K2) + 0.01;

r1r = 0.1;

r2f = 0.1;

r2r = 0.3;

r3f = 0.1;

r4f = 0.1;

%======

% Caculate reaction time

Tlink_R1 = -log(rand) / (r1f * C_FAKina);

Tlink_R2 = -log(rand) / (r1r * C_FAKa);

Tlink_R3 = -log(rand) / (r2f * C_FAKa);

Tlink_R4 = -log(rand) / (r2r * C_Scl);

Tlink_R5 = -log(rand) / (r3f * C_MGF);

Tlink_R6 = -log(rand) / (r4f * C_MGF);

%======

% Min time

t1 = min(Tlink_R1,Tlink_R2);

t2 = min(Tlink_R3,Tlink_R4);

t3 = min(Tlink_R5,Tlink_R6);

t4 = min(t1,t2);

t5 = min(t3,t4);

tmin = t5;

%======

% Go on

t = t + tmin;

%======

% Reaction happen

if tmin==Tlink_R1

C_FAKina = C_FAKina - 1;

C_FAKa = C_FAKa + 1;

elseif tmin==Tlink_R2

C_FAKina = C_FAKina + 1;

C_FAKa = C_FAKa - 1;

elseif tmin==Tlink_R3

C_Scl = C_Scl + 1;

elseif tmin==Tlink_R4

C_Scl = C_Scl - 1;

elseif tmin==Tlink_R5

C_Scl = C_Scl + 1;

elseif tmin==Tlink_R6

C_MGF = C_MGF - 1;

end

%======

TimeArry(i,1) = t;

C_R_Arry(i,1) = C_Scl;

C_M_Arry(i,1) = C_MGF;

C_FAK_Arry(i,1) = C_FAKa;

if t>12 && t<=24 % Mean value of Scl between 12 to 24 hours

m = m + 1;

total = total + C_Scl;

end

means = total / m;

end

F_Arry(j,1) = means;

end

% figure (1)

% plot(TimeArry,C_R_Arry)

% hold on

% axis([0,100,0,300]);

% set(gcf,'unit','centimeters','position',[10,5,12,8])

% xlabel('Time (h)');

% ylabel('The molecular number of Scleraxis');

% save

% set(gcf,'color','white');

% A=getframe(gcf);

% imwrite(A.cdata,'Fig1.png')

% saveas(gcf,'Fig1.fig');

F_Arry

**References**

1. Seo BM, Miura M, Gronthos S, et al. Investigation of multipotent postnatal stem cells from human periodontal ligament. *Lancet.* 2004; 364(9429): 149-155.

2. Dobo-Nagy C, Fejerdy P, Angyal J, et al. Measurement of periapical pressure created by occlusal loading. *Int Endod J.* 2003; 36(10): 700-704.

3. Gu Y, Bai Y & Xie X. Bite Force Transducers and Measurement Devices. *Front Bioeng Biotechnol.* 2021; 9(665081.

4. Cheng B, Liu Y, Zhao Y, et al. The role of anthrax toxin protein receptor 1 as a new mechanosensor molecule and its mechanotransduction in BMSCs under hydrostatic pressure. *Sci Rep.* 2019; 9(1): 12642.

5. Andreasen JO. Relationship between cell damage in the periodontal ligament after replantation and subsequent development of root resorption. A time-related study in monkeys. *Acta Odontol Scand.* 1981; 39(1): 15-25.

6. Cheng B, Wan W, Huang G, et al. Nanoscale integrin cluster dynamics controls cellular mechanosensing via FAKY397 phosphorylation. *Sci Adv.* 2020; 6(10): eaax1909.
